# Illuminating cancer therapy: The translational path of optogenetics

**DOI:** 10.1016/j.bioactmat.2026.04.019

**Published:** 2026-04-21

**Authors:** Bing Yang, Qiyi Feng, Chunxiu Xiao, Xiuli Zheng, Jingyao Chen, Shuwen Xiao, Zhichang Liu, Kai Xiao

**Affiliations:** a Laboratory of Precision Therapeutics, Department of Pulmonary and Critical Care Medicine, Institute of Respiratory Health and Multimorbidity, State Key Laboratory of Respiratory Health and Multimorbidity, Precision Medicine Key Laboratory of Sichuan Province, Frontiers Science Center for Disease-Related Molecular Network, West China Hospital, Sichuan University, Chengdu, 610041, China; b Tianfu Jincheng Laboratory (Frontier Medical Center), Chengdu, 610213, China

## Abstract

Tumor recurrence, metastasis, and therapeutic resistance remain major challenges in oncology, driving the need for advanced therapeutic strategies with improved precision and controllability. Optogenetics, which enables light-mediated regulation of cellular functions, has emerged as a promising modality for cancer therapy by offering unparalleled spatiotemporal precision. This capability allows dynamic control of intracellular signaling and transgene expression, enabling selective targeting of malignant cells while minimizing damage to surrounding tissues. However, clinical translation is hindered by key challenges, including inefficient *in vivo* delivery of optogenetic components, limited tissue penetration of activating light, and suboptimal performance of existing tools. Addressing these barriers requires a convergence of molecular engineering and materials science, wherein advanced biomaterials play a critical role in enabling gene delivery and overcoming tissue-penetration limitations in complex tumor environments. In this review, we provide a comprehensive oriented overview of optogenetics in oncology. We first analyze the molecular mechanisms and engineering principles of representative optogenetic tools, with a focus on LOV- and CRY2-based systems. We then highlight recent advances in biomaterial-assisted optogene delivery and light delivery strategies, emphasizing their material-dependent mechanisms that enable precise spatiotemporal control *in vivo*. Furthermore, we summarize emerging preclinical applications in cancer immunotherapy, gene regulation, and intracellular signaling control. Finally, we discuss key challenges in biosafety, kinetic optimization, and clinical scalability, and outline future directions that integrate optogenetics with functional materials and intelligent design to realize clinically viable platforms. This review aims to provide a framework for the development of clinically viable optogenetic platforms for next-generation cancer therapy.

## Highlights

• This review provides a mechanistic overview of optogenetic strategies for cancer therapy.
• The performance of each system is defined from molecular mechanism to kinetic behavior.
• Gene and light delivery strategies are systematically examined to achieve *in vivo* optogenetic activation.
• Material-based noninvasive light delivery approaches are summarized.
• This work offers practical guidance for advancing optogenetic platforms toward translational oncology.

## Graphical abstract

**Figure:**
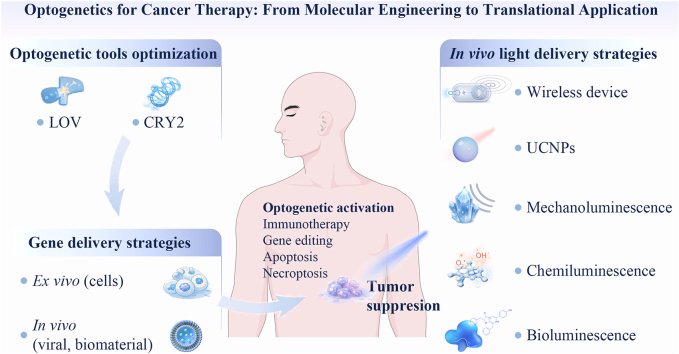

## 1 Introduction

Despite substantial progress in conventional cancer treatments including surgical resection, chemotherapeutic regimens, precision radiotherapy, and molecularly targeted agents [1,2], clinical outcomes are frequently limited by tumor recurrence, metastatic dissemination, and acquired therapeutic resistance [3]. These challenges, compounded by systemic toxicity and off-target effects, underscore the urgent need for therapeutic modalities with enhanced spatial precision and temporal control.

Optogenetics, an interdisciplinary biotechnology that integrates optical modulation with genetic engineering, has emerged as a transformative strategy to overcome these clinical constraints [4]. Unlike conventional systemic therapies such as chemotherapy or radiotherapy, which operate through diffusible mechanisms and often lack spatial selectivity, optogenetic systems enable light-controlled regulation of cellular functions with high spatiotemporal resolution. This capability allows targeted modulation of malignant cells or specific components within the tumor microenvironment (TME) while minimizing damage to surrounding healthy tissues. Moreover, optogenetics offers a unique degree of molecular programmability: by coupling photosensitive modules with diverse effector domains, cellular behaviors including gene expression, protein-protein interactions, and signal transduction can be dynamically and reversibly tuned in a user-defined manner [[5], [6], [7]]. When integrated with advanced material systems such as implantable devices or light-converting nanoparticles, optogenetic platforms further enable non-invasive or minimally invasive activation in deep tissues. Collectively, these features position optogenetics as a highly versatile and controllable therapeutic strategy with the potential to overcome key limitations of existing cancer treatments.

**Fig. 1:**
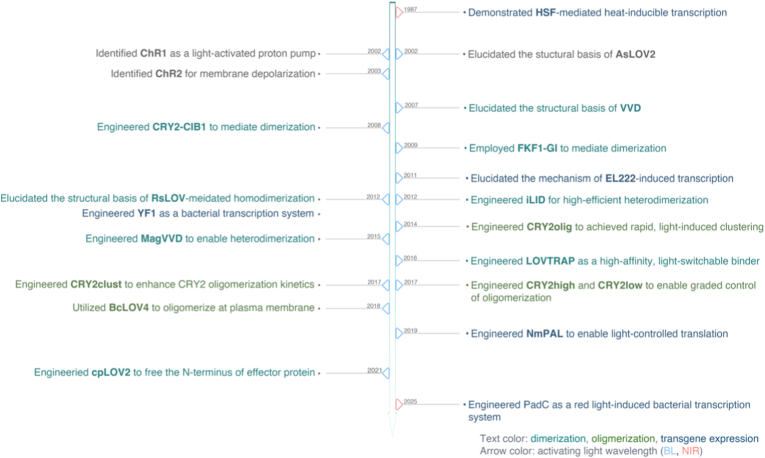
Historical progression of optogenetic modules and their mechanistic advances.

Over the past decades, the field of optogenetics has advanced rapidly, driven by the continual development of diverse optogenetic tools (OTs) that have expanded the therapeutic repertoire in oncology (Fig. 1). Initially pioneered in neuroscience to modulate neuronal activity with opsins-based tools [[8], [9], [10], [11], [12], [13]], optogenetics have since been adapted for oncological applications, opening new avenues for immunotherapy, gene therapy, and light-induced cytotoxicity [[14], [15], [16], [17]]. A particularly significant advance is the emergence of non-opsin OTs, which utilize modular photoreceptors fused to effector proteins. These tools enable precise reprograming of the TME, fine-tuning of immune responses, and targeted induction of cancer cell death.

Despite these promises, the clinical translation of optogenetics faces three major hurdles: (1) efficient *in vivo* delivery of optogenes to target cells, which is hampered by vector tropism, tumor heterogeneity, and immune evasion [18]; (2) limited tissue penetration of visible light required to activate conventional OTs, which restricts their application to superficial tumors unless coupled with advanced light-delivery modalities; and (3) the need to optimize the molecular properties of OTs, such as photosensitivity, switching kinetics, and biocompatibility in mammalian systems, to ensure robust performance under complex physiological conditions [19,20].

Opsin-based tools, which primarily modulate cellular activity through light-gated ion flux, have been extensively reviewed in neuroscience and show promise in regulating membrane potential, ion homeostasis, and GPCR signaling [14,16,21]. However, their application in oncology remains relatively constrained by their predominant membrane-level mode of control. In contrast, non-opsin OTs offer more diverse regulatory modalities, enabling direct and programmable manipulation of intracellular processes. Consequently, non-opsin OTs have emerged as a rapidly developing yet predominantly preclinical field in oncology [15,[22], [23], [24], [25], [26], [27]]. Among these, LOV- and CRY2-based systems are the most extensively developed, providing a broad spectrum of engineered variants and regulatory mechanisms. We acknowledge that red- and far-red-responsive platforms (e.g., PhyB–PIF and bacterial phytochrome systems) offer important advantages for tissue penetration and thus hold significant translational potential. However, such systems often involve more complex photochemistry, require exogenous chromophore supplementation, and have seen comparatively limited application in cancer therapy to date. Given that this review aims to provide a function-oriented perspective on optogenetic strategies for cancer therapy and to inspire potential applications across diverse tumor contexts, we focus primarily on the more mature and widely adopted LOV- and CRY2-based platforms, while summarizing red/far-red systems in Table 1 to reflect their emerging relevance.

**Table 1:** *In vitro* optogenetic system in cancer therapy.

OT	Regulation mode	Design	Cell line(s)	Target	Signaling mechanism	Ref.
AsLOV2	POI translocation	NES-mCh-AsLOV2-NLS-YAP	MKN28	YAP	Light exposure unfolds the Jα helix to expose aNLS, driving YAP nuclear import.	[84]
AsLOV2	POI assembly	Ecad (D134)-AsLOV2-Ecad (D136)	MDA-MB-231	E-cadherin	Light-driven destabilization of engineered E-cadherin calcium-binding sites disrupts Ca^2+^ affinity and impairs cell-cell adhesion.	[85]
AsLOV2	POI translocation	LOV2-BAX-TMD	HeLa	BAX	OS induces BAX conformational activation and mitochondrial translocation, resulting in membrane permeabilization.	[86]
AsLOV2	Ca^2+^ influx	ORAI1-LOV2	293T	ORAI1	Incorporation of a LOV2 domain into ORAI1 enables light-gated Ca^2+^ influx.	[87]
iLID	POI translocation	LOV2-SsrA-CAAX iSH2-SspB	A375	PI3K	Optical targeting of PI3K to the PM drives PIP3 hyperactivation and TNFAIP8 upregulation, conferring drug resistance in melanoma.	[88]
iLID	POI translocation	LOV2-CAAX DH-PH-SspB	RPE1	RhoA	Light-mediated PM translocation of RhoGEFs induces retraction at low levels, and protrusion at high levels via Cdc42 activation and RhoA sequestration.	[89]
PhoBIT1	ligand release	SspB (LOV2)-TM SsrA-PAR	Hela	PAR	Light-induced dissociation of SsrA and SspB releases the tethered ligand, allowing it to engage the receptor and trigger PAR activation.	[90]
LOVTRAOP	POI release	LOV@-MTS Zdk1-NOTCH1	MCF7 MDA-MB-468	NOTCH	Light-triggered release of the NOTCH1 intracellular domain from the PM, enabling its nuclear translocation and transcriptional activation.	[91]
YF1/FixJ	transcription	pDawn-IFNγ	*Lactococcus lactis*	IFNγ expression	An upconversion nanoparticle-based optogenetic system enables NIR-mediated induction of IFNγ expression.	[58]
CRY2-CIB1	POI translocation	p53-CRY2 NLS-CIB1	Hela	Autophagy	Light-controlled p53 nuclear translocation induces autophagy.	[92]
CRY2-CIBN	POI translocation	CIBN-CAAX CRY2-iSH2	A549	PI3K	OS recruits cytosolic PI3K to the PM, driving PIP3 production, Akt activation, E-cadherin downregulation, and epithelial-mesenchymal transition to promote metastasis.	[93]
CRY2-CIB1	protein interaction	dCas9-CIB1 CRY2-AD	5637 UMUC-3	CRISPR	CRY2-CIB1 heterodimerization recruits active domain (AD) to dCas9, initiating transcription.	[94]
CRY2	protein interaction	C-RAF-CRY2	293T	MAPK	Optogenetic induction of C-RAF dimerization to activate downstream MAPK signaling.	[95]
CRY2	protein interaction	PHR-Caspase8	Hela	Caspase8	Light-induced oligomerization of CRY2-fused caspase8 triggers its self-cleavage and activation of caspase3, inducing apoptosis.	[96]
CRY2PHR	POI translocation and interaction	Lyn11-MyD88ΔTIR-CRY2-mCh	Hela	MyD88	Light-induced MyD88 oligomerization at the PM selectively activates NF-κB, IRF7, and cytokine production without off-target signaling.	[97]
CRY2PHR	Protein interaction	MLKL-PHR-GFP	Hela	Necroptosis	Light-triggered MLKL oligomerization induces RIPK3-independent necroptosis, exerting antitumor effects and enhancing antitumor immunity via DAMPs release.	[98]
PHY-PIF	POI translocation	PHY-CAAX PIF-SOScat	NIH 3T3s	Ras-Erk	Red light induces the recruitment of SOS2 to the PM for Ras activation, a process disruptable by B-Raf mutations or inhibitors.	[99]

In this review, we systematically examine the molecular mechanisms of OTs with clinical promise, alongside strategies for their targeted delivery and non-invasive activation. We critically evaluate recent preclinical advances in optogenetic oncology, with a focus on applications in immunotherapy, gene editing, and cancer cell death induction. Furthermore, we dissect persistent challenges and propose interdisciplinary solutions to bridge the gap between bench and bedside. By contextualizing optogenetics within the framework of precision medicine, we aim to catalyze the development of next-generation, light-controllable therapeutic platforms capable of redefining cancer treatment.

## 2 OT categories for cancer therapy

OTs are modular molecular systems comprising two core functional units: a photosensitive module (PSM) and an effector module (EM). Upon exposure to light of specific wavelength, the PSM absorbs photons and initiates a photochemical reaction that induces a conformational change. This structural rearrangement is then transmitted to the EM, thereby modulating its biological activity, such as enzymatic function or protein-protein interactions. The photosensitivity of an OT is primarily governed by the embedded chromophore within the PSM, while the EM executes its function through intra- or intermolecular interactions, such as electrostatic forces or hydrophobic binding. For translational oncology, OTs must meet stringent criteria. They must be activatable by long-wavelength light (e.g., near-infrared, NIR) to overcome the poor tissue penetration of conventional short-wavelength visible light (400–500 nm). Slow off-switch kinetics are also crucial to reduce the frequency of light administration, a significant advantage for treating deep-seated tumors. Additionally, single-wavelength control is preferred for clinical practicality, as multi-wavelength systems complicate *in vivo* applications [28]. Finally, OTs should rely on endogenous cofactors naturally available in mammalian cells, avoiding the need for exogenous cofactors that would complicate clinical translation [29].

### 2.1 OTs suitable for cancer therapy

The diversity of OTs stems from several chromophore families, including retinal-based rhodopsins, flavin-based systems such as LOV and CRY2, and biliverdin IXα–based bacterial phytochromes (BphPs). Among these, LOV- and CRY2-based OTs are particularly promising for clinical translation due to their superior photosensitivity and compatibility with mammalian physiology. This section critically evaluates LOV- and CRY2-based OTs that meet these translational benchmarks, supplemented by a discussion of emerging photothermal systems.

#### 2.1.1 LOV-based OTs

The light-oxygen-voltage (LOV) domain, a widespread photosensory module in plants and microorganisms, has emerged as a cornerstone of OT development owing to its modular structure, high photosensitivity, and compatibility with mammalian systems [20]. Structurally, the LOV domain comprises a flavin mononucleotide (FMN) chromophore, a Per-ARNT-Sim (PAS) sensory domain, and a C-terminal Jα-helix. FMN, the smallest chromophore among known OTs, exhibits high photosensitivity as a derivative of endogenous riboflavin (vitamin B2). Its structure consists of an isoalloxazine ring (the photoactive site), a ribitol moiety, and a phosphate group.

**Fig. 2:**
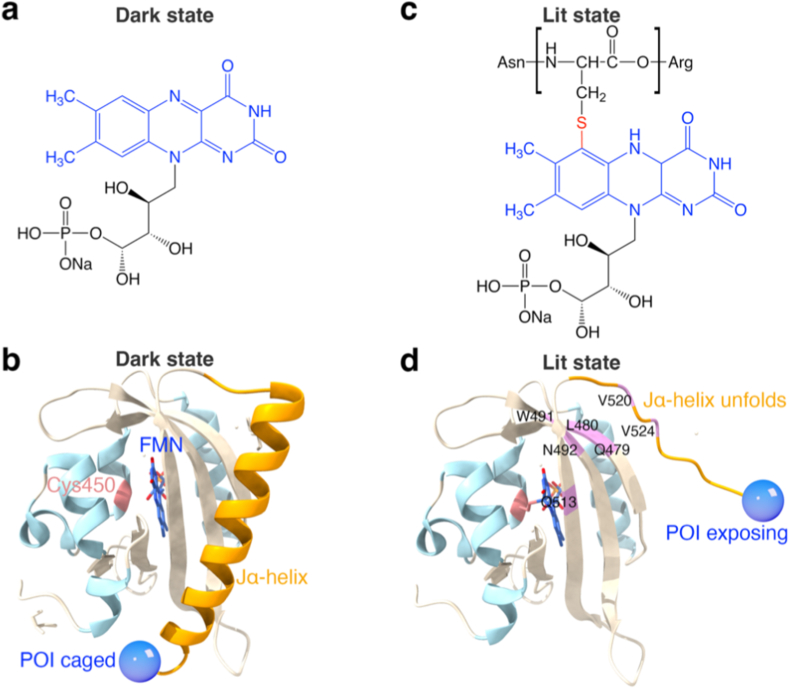
Mechanism of LOV2 photoactivation and Jα-helix unfoldi**ng. (a, b)** Dark state. In the absence of BL, the FMN chromophore is in its oxidized form. The Jα-helix is tightly packed against the LOV core, sterically caging the fused POI. **(c, d)** Lit state. BL irradiation promotes FMN into a triplet excited state, resulting in the formation of a covalent Cys–FMN adduct (between the C4a atom of FMN and the S atom of C450). This adduct induces conformational rearrangements that propagate through the LOV core, leading to the unfolding of the Jα-helix and the exposure of the POI. Key residues critical for this conformational transmission are highlighted in (d).

The photoactivation of LOV follows a multi-step mechanism: (1) Blue light (BL, 450–495 nm) absorption: The extended π-conjugation system of the isoalloxazine ring enables BL absorption, promoting a π–π∗ transition to the singlet excited state. (2) Excited-state reactivity: The local environment of LOV promotes spin-orbit coupling of π∗ electron, facilitating intersystem crossing to the longer-lived triplet state [30]. This enhances FMN's reactivity, leading to homolytic cleavage of the bond at the C4a position, generating a reactive FMN radical. (3) Covalent adduct formation: The electrophilic C4a carbon forms a reversible thioether bond with a conserved cysteine residue (typically C450), yielding a flavin-cysteinyl adduct (C4a-S-Cys). (4) Signal transduction: This covalent modification triggers conformational changes propagated through a conserved residue pathway (FMN-Q513-N492-L480-W491-Q479-V520-A524) [31] and a Helix-Hairpin-Helix (H-H-H) structure [32]. In the dark state, the Jα-helix is packed against the PAS core via hydrogen bonding and hydrophobic interactions (Fig. 2a and b). Light-induced adduct formation disrupts these interactions, exposing hydrophobic residues (e.g., leucine, isoleucine, phenylalanine) and unfolding the Jα-helix [33]. Upon light withdrawal, the C4a–thiol adduct spontaneously dissociates, allowing the Jα-helix to refold to its native conformation (Fig. 2c and d) [32,34]. Below, we summarize key engineered LOV variants with translational potential in oncology.

(1) AsLOV2

**Fig. 3:**
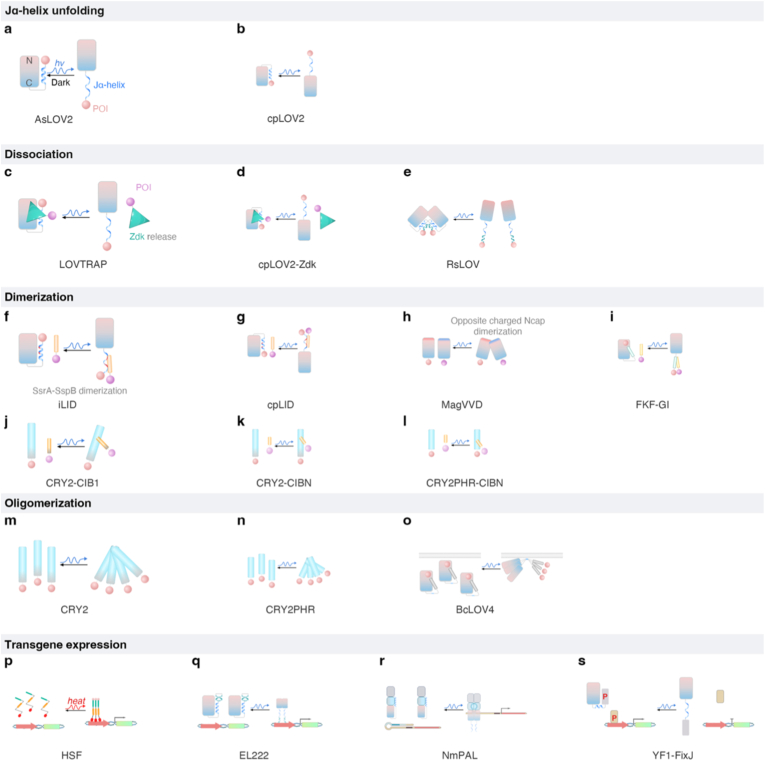
Molecular mechanisms of representative OTs with translational potential. OTs are categorized based on their light-induced functional outputs. **(a, b) Jα-helix unfolding.** BL triggers conformational changes in LOV domains, leading to unfolding of the Jα-helix and exposure of the fused POI. AsLOV2 uncages the C-terminus, while cpLOV2 enables N-terminal control. **(c**–**e) Dissociation.** BL-induced structural rearrangements disrupt protein–protein interactions, resulting in release of bound components. **(f**–**l) Heterodimerization.** Light exposure induces conformational changes that expose interaction interfaces, enabling binding between specific partners (e.g., CRY2–CIBN, LOV-based systems). Note: The CRY2-CIB1 interaction is mediated by the N-terminal CRY2PHR and CIBN domains; the C-terminal CCT domain of CRY2 is dispensable for both CRY2PHR-CIBN dimerization and CRY2PHR oligomerization. **(m**–**o) Oligomerization.** Light stimulation promotes homodimerization or higher-order clustering, facilitating signal amplification or membrane recruitment. **(p**–**s) Transgene expression.** Light regulates gene expression through transcription factor assembly, phosphorylation control, or modulation of mRNA structure, thereby influencing transcriptional or translational outputs. Representative systems are selected based on their mechanistic diversity and translational relevance.

AsLOV2, derived from *Avena sativa*, represents the basic framework of LOV domain and is commonly utilized as a photocage to regulate the release of protein of interest (POI). Typically, the POI is fused to the C-terminus of the Jα-helix. In the dark state, the folded Jα-helix sterically blocks the POI's functional domain. BL illumination triggers Jα-helix unfolding, liberating the POI for functional activity (Fig. 3a). AsLOV2 exhibits advantages such as rapid photoactivation, reversible switching kinetics, compact size, and minimal cytotoxicity, making it highly amenable to gene vector packaging and *in vivo* applications. However, it is also subjected to certain limitations. Spontaneous unfolding of Jα-helix, as well as the fusion of large POIs that destabilize the Jα-PAS interaction, can increase the basal activity in the dark state [35]. Engineered variants with improved dark-state stability have been developed to minimize leakiness, which is critical for translational applications requiring high specificity.

(2) LOVTRAP

As an evolved version of photocage, the LOV2 Trap and Release of Protein (LOVTRAP) system exhibits lower leakiness than AsLOV2. It employs a two-component architecture: the LOV2 domain and a high-affinity binding partner, Zdk [36]. In the dark, Zdk binds tightly to both the LOV2 domain and Jα-helix, effectively sequestering the fused POI and preventing its functional engagement. Upon illumination, conformational changes in the LOV2 core and unwinding of the Jα-helix disrupt the LOV2–Zdk interaction, releasing the POI for downstream functions (Fig. 1c). LOVTRAP achieves rapid activation (<1 s), tunable deactivation, and >100-fold reduction in basal activity compared to AsLOV2. Notably, the LOV2 domain, rather than Zdk, should serve as the anchored component [37].

(3) iLID

The improved light-induced dimer (iLID) system expands AsLOV2 functionality by embedding a seven-residue SsrA peptide into the Jα-helix. Due to sequence similarity and compatible helical propensity, the incorporation of SsrA preserves both the structural stability of the Jα-helix and the binding functionality of the SsrA peptide. In the dark state, the SsrA motif remains sequestered. BL illumination triggers Jα-helix unfolding and exposes SsrA, enabling high-affinity binding to its cognate partner SspB (Fig. 3f) [38]. This design enhances the binding affinity between the Jα–SsrA chimera and SspB by approximately 8-fold [24], facilitating spatiotemporally precise and reversible control of protein-protein interactions. Rational mutagenesis of PAS-domain (e.g., R73Q) that modulate Jα-helix stability further suppresses dark-state leakage and improves the dynamic range by 58-fold [39]. Due to its prokaryotic origin, the SsrA peptide and SspB minimizes off-target interactions in mammalian proteins, making iLID particularly suitable for controlling intracellular signaling, such as light-inducible assembly of chimeric antigen receptors (CARs) [17].

(4) cpLOV2

Circularly permuted LOV2 (cpLOV2) expand the utility of AsLOV2-based OTs by enabling optical regulation of POIs with functional N-terminal domains [40]. Due to the compact structure of LOV2 and the proximity of its N- and C-termini (<25 Å), the Jα-helix can be repositioned at the N-terminus through circular permutation, creating new caging surfaces (Fig. 3b). cpLOV2 can function as a photocage, a dissociation switch (Fig. 3d), or an optical heterodimerization system. For instance, circular permutated LID (cpLID) incorporates SsrA peptide into the N-terminal Jα-helix, mimicking the kinetics of iLID (Fig. 3g), broadening the applicability of LOV [40,41].

(5) VVD

*Neurospora crassa* Vivid (VVD) is one of the smallest known LOV-domain photoreceptors that uses flavin adenine dinucleotide (FAD) as chromophore to mediate light-dependent homodimerization. BL induces structural reorganization in wild-type VVD (WT-VVD), exposing hydrophobic residues and dimerization motifs in the Ncap that stabilize homodimer formation [42]. The WT-VVD exhibits slow photocycle kinetics (t_1/2_ = 2.8 h) and weak dimerization ability. The engineered variants MagVVD overcome these limitations by introducing oppositely charged amino acids into the neutral Ncap [43]. In MagVVD, positively charged pVVD (I52R/M55R) and negatively charged nVVD (I52D/M55G) suppress homodimerization via electrostatic repulsion while enabling controlled heterodimer assembly (Fig. 3h). Due to overstabilization of the C4a-flavin adduct, VVD display slow deactivation kinetics. Mutations within PAS domain can enhance dimerization efficiency by op to 700%, with dissociation times adjustable from minutes to hours [43,44]. These properties are advantageous for transcriptional and translational control applications where prolonged activation is desired [45].

(6) FKF1-GI

Flavin-Binding Kelch Repeat F-Box 1 (FKF1) is a light-responsive protein from *Arabidopsis thaliana* implicated in photoperiodic regulation. It comprises three functional domains: a F-box motif, Kelch Repeats, and a LOV domain. BL-induced conformational changes in the LOV domain exposes Kelch Repeats and enabling selective heterodimerization with GIGANTEA (GI) (Fig. 3i). A major advantage of this system is the remarkable stability of its C4a-flavin adduct state, which sustains FKF1–GI dimers for over 1.5 h after light withdrawal, reducing the need for continuous illumination [46]. However, it suffers from several limitations, including slow activation kinetics, potential nonspecific binding at high concentrations, and a constitutive nuclear localization signal (NLS) that restricts its use to the nucleus. Cytoplasmic applications require strategic NLS mutagenesis or incorporation of nuclear export signals (NES) [46].

(7) BcLOV4

BcLOV4, from *Bacillus cereus*, is a single-component photoreceptor that translocate from cytoplasm to the plasma membrane (PM) upon BL exposure. Its LOV domain contains a cationic amphipathic helix (AH), which binds anionic phospholipids, enabling PM recruitment and nanocluster formation (Fig. 3o) [[47], [48], [49]]. This enables modulation of oligomerization-dependent cascades such as EGFR, RAS and RhoA signaling [47,50,51]. As a single-component system, BcLOV4 exhibits low basal activity and a broad dynamic range. However, its activation is transient due to feedback inhibition or photoadaptation, often requiring pulsed illumination for sustained signaling output [51].

(8) RsLOV

*Rhodobacter sphaeroides* LOV (RsLOV) represents a unique subclass of OTs that operates through ‌inverse photoswitching. Unlike typical LOV-based systems that tundergo light-induced dimerization, RsLOV exists as a homodimer in the dark and dissociates into monomers under BL illumination (Fig. 3e). Structurally, RsLOV contains a N-terminal A’α helix (Ncap) and a C-terminal helix-turn-helix (HTH) motif. In the dark state, the dimer is stabilized by hydrophobic packing and hydrogen bonding between the N- and C-terminal extensions. Upon optical stimulation (OS), the conformational change disrupt the interface, leading to dimer dissociation [52]. This unique light-off mechanism enables RsLOV to function as a reversible dissociation switch, offering potential for applications requiring light-controlled protein release or inactivation.

(9) EL222

EL222 is a compact LOV-based optogenetic transcription factor (222 amino acids) that operates through light-induced homodimerization. It contains a LuxR-type HTH DNA-binding domain located downstream of Jα-helix. In the dark state, the Jα-helix sterically blocks the DNA-binding interface of the HTH domain [53]. Light-induced conformational changes trigger Jα-helix unfolding and subsequent homodimerization through the exposed HTH domains. This enables sequence-specific binding to the synthetic promoter C120 (pC120), initiating transcription of downstream genes (Fig. 3q). Despite its therapeutic potential, EL222 suffers from several limitations including promoter leakiness [54], limited dynamic range [55], and rapid deactivation speed (within seconds). Current optimization efforts focus on two main strategies: coupling pC120 with orthogonal amplification systems, such as the yeast Gal4 transcription circuit, to enhance output signals [56]; and introducing dimer-stabilizing mutations to prolong the active state [57].

(10) YF1-FixJ transcription system

The YF1-FixJ system is a bacterial two-component optogenetic platform that regulates transcription through a phosphorelay mechanism [58,59]. It consists of two core components: YF1, a chimeric histidine kinase combining an N-terminal LOV domain from *Bacillus subtilis* YtvA with a C-terminal histidine kinase (HK) domain, and FixJ, a response regulator transcription factor from *Bradyrhizobium japonicum* (Fig. 3s) [60]. In the dark state, the HK domain undergoes autophosphorylation and transfers the phosphate group to FixJ. Phosphorylated FixJ subsequently binds the FixK2 promoter to initiate transcription. Upon BL illumination, conformational changes in the LOV domain suppresses YF1 kinase activity, thereby inhibiting FixJ phosphorylation and downregulating target gene expression.

To invert the transcriptional output, an engineered system called pDawn was developed by introducing an inversion element, λ phage repressor (cl) from *Escherichia coli*. In the dark state, the cl expression, driven by PFixK2, suppresses the strong λ pR promoter, thereby inhibiting transcription of downstream genes. BL illumination relieves this repression, resulting in light-induced transcriptional activation [59]. This redesigned system exhibits high photosensitivity, robust maximal expression, and negligible basal activity, making it particularly suitable for *ex vivo* applications. However, its requirement for prolonged OS (>2 h) limits its use in deep tissue setting. Additional constraints include inherent incompatibility with eukaryotic cellular machinery, necessitating further engineering for cross-kingdom implementation.

(11) NmPAL

NmPAL is a prokaryotic OT that regulates gene expression at the translational level by controlling ribosome access to ribosome-binding sites (RBS). In bacteria, translation initiation requires precise alignment of the ribosome with the start codon via the Shine-Dalgarno (SD) sequence (AGGAGG) within the mRNA. When the SD region is sequestered within a hairpin structure formed by a complementary sequence at the 5′ untranslated region, ribosomal binding is sterically inhibited, thereby suppressing translation. Derived from *Nakamurella multipartita*, the LOV-domain protein NmPAL modulates translation by reversibly unfolding this hairpin to expose the RBS upon light activation (Fig. 3r).

Structurally, NmPAL contains a PAS-ANTAR-LOV-Jα arrangement and undergoes light-dependent homodimerization [61]. The ANTAR domain regulates translation by specifically binding to mRNA adapter sequences within the hairpin, notably the motif-3 adapter (GGTTGAAGCAGACGACC). In the dark state, the Jα-helix sterically blocks the ANTAR RNA-binding interface. BL-induced homodimerization of the ANTAR domain licenses the motif-3 adapter to bind and induce mRNA loop formation. This breaks the hairpin structure, thereby exposing the RBS and enabling translation initiation [62]. This system exhibits an extremely low basal expression and a remarkably broad dynamic range (>1000-fold), making it a valuable tool for multi-layer genetic circuits requiring precise, rapid control over protein synthesis.

#### 2.1.2 CRY2-based OTs

Cryptochrome 2 (CRY2) represents a distinct class of BL-activated OTs, differing fundamentally from LOV domains in structure and activation mechanism [63]. Upon BL stimulation, CRY2 undergoes both dimerization and oligomerization (Fig. 3j–n). Structurally, it comprises a photosensitive photolyase-homologous region (PHR) domain and a C-terminal extension (CCT). BL induces conformational rearrangements within the PHR domain, exposing the interaction interface that mediates CRY2-CRY2 association via hydrophobic interactions and hydrogen bonding [64].

Although CRY2 also utilizes FAD as a chromophore, its photocycle differs mechanistically from that of LOV domains. BL excites FAD to a singlet excited state (*vs.* LOV's triplet state), initiating an intramolecular electron transfer cascade via nearby aromatic residues (e.g., tryptophan and tyrosine). This redox reaction reduces FAD to FADH^−^ state, triggering charge redistribution and structural reorganization within the PHR. Concurrently, BL induces partial unfolding of the N-terminal α-helical segment and C-terminal β-folded region, further facilitating higher-order oligomerization and cluster assembly.

(1) CRY2 oligomerization

Protein homo-oligomerization governs diverse cellular functions, including membrane receptors activation, kinases signaling, and transcriptional activity [65]. Among natural photoreceptors, CRY2 uniquely enables direct light-induced oligomerization via specific α-helices (α13, α14, α19, and α20) within the PHR domain [64,66]. However, native CRY2 exhibits suboptimal clustering efficiency due to steric hindrance from CCT and a requirement for high concentration (Fig. 3m) [64,[67], [68], [69]].

To mitigate these limitations, engineered CRY2 variants with improved oligomerization properties have been developed. For example, the E490G mutation (CRY2olig) enhances clustering efficiency by approximately 100-fold and extends the active-state half-life from 6 min to 23.1 min, enabling sustained signaling post-illumination [68,70]. In contrast, CRY2clust, generated by fusing CCT with a 9-amino-acid peptide (ARDPPDLDN), exhibits rapid on/off switching kinetics within seconds [71]. This variant operates effectively at low light intensities and shorter OS duration, making it ideal for modulating shallow tumors or cortical tissue [72,73].

(2) CRY2-CIB1 dimerization

The CRY2 system supports both homo- and hetero-assemblies, each with unique biophysical properties. Wild-type CRY2 (WT-CRY2) undergoes dynamic clustering under BL, forming oligomers ranging from dimers to tetramers. Notably, clustering efficiency is markedly enhanced when the POI is fused to the C-terminus of CRY2, likely due to reduced steric hindrance and enhanced conformational flexibility [65,74]. Additionally, CRY2 specifically heterodimerizes with its natural binding partner CIB1, a basic helix-loop-helix (bHLH) protein from *Arabidopsis thaliana*. This interaction is mediated by the bHLH of CIB1 and specific lysine residues (Lys116, Lys118, and Lys146 residues) within the PHR domain (Fig. 3j–l) [75,76]. A truncated CRY2 variant (containing only the PHR domain, aa 1–498) exhibits a 12-fold increase in reporter activity compared to full-length CRY2 (aa 1–612), which improves accessibility to the CIB1-binding interface and reduces autoinhibitory effects [77].

#### 2.1.3 Heat shock factor-derived OTs

Beyond exogenous optogenetic systems, endogenous eukaryotic components such as the heat shock factor (HSF) provides considerable potential for optogenetic engineering [[78], [79], [80], [81], [82]]. HSF is a common mammalian transcription factor that trimerizes under thermal stress and binds to heat shock elements (HSEs), initiating the transcription of heat shock proteins (HSPs). The activation mechanism comprises three key steps: (1) Under basal conditions, HSF monomers bind to HSPs and keep in an inactive state. (2) Under thermal stress, cellular proteins unfold or misfold, exposing hydrophobic regions. HSPs dissociate from HSF to bind these hydrophobic regions, initiating protein repair or degradation. The released HSF monomers, which contain a leucine-rich zipper, undergo self-trimerization via hydrophobic surfaces. (3) Trimerized HSF exposes its NLS, translocates to the nucleus, binds HSE promoters, and activates HSP transcription. Once the stress is alleviated, HSF rebinds to newly synthesized HSP and returns to its inactive state (Fig. 3p). This endogenous thermal-response system presents several advantages: inherent biocompatibility with minimized cytotoxicity, signal amplification through natural cascades, and reduced genetic payload. Optogenetic adaptation enables transgene regulation through biomaterial-mediated photothermal conversion. Thermal activation can be achieved via NIR, focused ultrasound (FUS), or magnetic stimulation, offering versatility in experimental and clinical settings [83].

Optogenetics enables precise spatiotemporal control over subcellular organelles and signaling networks, providing unprecedented insights into tumor pathogenesis and holding significant potential for clinical translation (Table 1).

### 2.2 Kinetic behavior and optimization of OTs

The optimal therapeutic application of OTs requires rapid activation (on the order of seconds) and tunable deactivation kinetics to enable precise temporal control while minimizing light exposure. The switching behavior of OTs is diverse and governed by multiple parameters, including light intensity, expression level, local temperature, catalytic architecture, amino acid microenvironment, and the stability of the light-activated conformation. Most FMN/FAD-based systems exhibit rapid activation (seconds) due to excellent chromophore photoreactivity [100]. In contrast, deactivation rates vary widely (from minutes to hours) and are primarily influenced by the lit-state stability [101]. In therapeutic setting, slower deactivation is often advantageous as it reduces the frequency and duration of OS. In LOV domains, deactivation is governed by three principal molecular processes: (1) the kinetics of N5 deprotonation, which accelerates adduct decay by destabilizing the flavin N5–H bond; (2) steric modulation of FMN-Cys108 adduct by residues surrounding the isoalloxazine ring; and (3) electronic effects that disfavor reduced-flavin states, promoting thermal reversion. These processes are regulated by specific residues (e.g., Ile74 and Ile85 in VVD) through solvent/base accessibility via flavin-adjacent channels and stereo-electronic interactions within the chromophore-binding pocket [44].

**Fig. 4:**
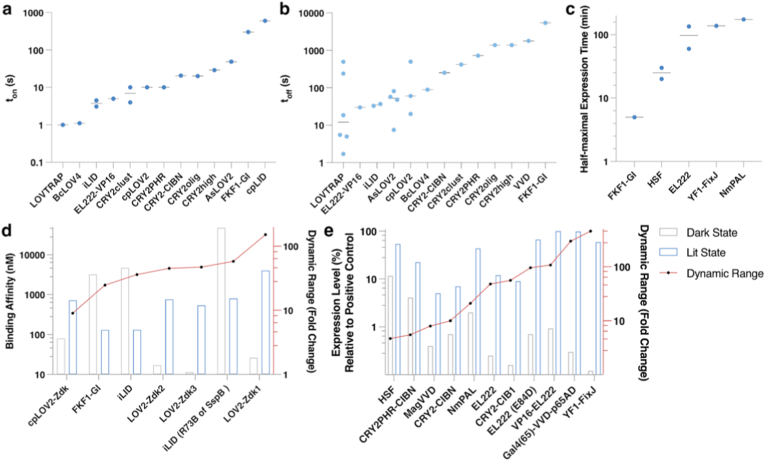
Comparison of optogenetic tools across kinetic, binding, and transcriptional performance metrics. **(a**–**b)** Kinetic profiles of OTs for controlling protein-protein interactions, showing (**a**) activation kinetics (*t*_on_, time to half-maximal activation upon illumination) and (**b**) deactivation kinetics (*t*_off_, time to half-maximal reversal after light withdrawal). **(c)** Half-maximal response times for optogenetically regulated gene expression. **(d)** Comparison of binding affinity and dynamic range for optogenetic protein–protein interaction systems. **(e)** Light-dependent transcriptional output of representative optogenetic expression systems. Bars indicate relative expression levels in the dark and illuminated states, and the line represents the resulting dynamic range. The summarized performance metrics are compiled from representative studies in the literature. These values are intended to provide a comparative overview rather than strictly standardized quantitative measurements. In panel (e), protein expression levels are presented as relative values, with the maximum expression level in each individual study set as 100%. Ref. (**a**) [37,40,41,46,49,54,65,68,77,102,103]; (**b**) [40,41,46,49,54,65,68,77,102]; (**c**) [37,39,46,54,62,77,104]; (**d**) [37,39,41,46]; (**e**) [18,19,40,59,65,68,77,104,105].

In the context of signal transduction modulation, LOV/CRY2-based OTs typically activate within 1 min under relatively low BL intensities (10 μW/cm^2^–10 mW/cm^2^) (Fig. 4a). Their deactivation speeds (t_off_), however, span in a broader range (10–1000 s) (Fig. 4b). Systems such as LOVTRAP, iLID and cpLOV2 exhibit both rapid activation and fast deactivation, making them particularly well-suited for signaling events that rely on transient, contact-initiated interactions, such as MAPK cascades and Src-family kinase signaling. Notably, CRY2-based clustering systems typically exhibit rapid activation but slower dissociation, supporting sustained signaling output.

For biosynthesis systems, longer illumination (from minutes to hours) is typically required to achieve sufficient protein levels (Fig. 4c). Among these, the HSF photothermal system enables robust transcriptional activation within approximately 20 min, outperforming EL222 and YF1-based systems that requires multi-hour exposure, highlighting its practical advantage for therapeutic use.

### 2.3 Leakage and dynamic range

Effective optogenetic modulation requires precise control of output signals (e.g., protein translocation, gene expression) to surpass activation thresholds while minimizing background activity. Although complete elimination of dark-state leakage remains challenging due to inherent conformational equilibria in most OTs, strategic protein engineering can significantly enhance their dynamic range. In AsLOV2-based systems, Jα-helix docks onto the A’α-helix in the dark state, suppressing background activity. In the dark state, approximately 1.6% of the Jα-helix remains undocked, which increases to 91% upon OS [106]. However, fusion of an effector domain may destabilize this interaction through competitive binding to the LOV domain [35,107]. Common strategies to stabilize the dark-state docking include mutations (e.g., G528A, N528E, I532A, D419K) that enhance helix propensity, introduce new tertiary interactions, or improve electrostatic complementarity [35,39]. For instance, a C-terminal phenylalanine substitution in LOV2-SsrA enhanced its switching dynamic range by 2- to 8-fold [38]. In light-induced clustering OTs (e.g., EL222, CRY2, VVD), leakage often stems from dark-state clustering driven by hydrophobic interactions or electrostatic attractions. Introducing larger, neutral amino acids at these positions can sterically hinder such interactions. For example, in the CRY2-derived Ca^2+^ modulator (AtCRY2), electrostatic interactions within the protrusion loop (Lys268-Leu286) promote self-association, leading to dark-state dimerization and unintended Ca^2+^ influx [72]. Mutations such as E279A and E281A reduce these interactions, while substituting smaller residues with bulkier ones (e.g., G280W) introduces steric hindrance, effectively preventing self-association [72].

The dynamic range of an OT refers to the fold change between its maximal and basal output levels under OS, which critically determines the therapeutic efficacy. This parameter is governed by several interconnected factors. Intrinsic photochemical properties include photosensitivity, activation/deactivation kinetics, and photostability. Light stimulation parameters also play a key role, as higher intensity enhances activation but increases the risk of phototoxicity. Cellular context is another important consideration, given that performance varies across cell types and often requires optimizations beyond standard HEK293T models [108]. Finally, Output equilibrium must be carefully balanced: achieving a high dynamic range must be weighed against maintaining sufficient maximum output to reach therapeutic thresholds. Consequently, the background activity and output levels of various OTs can vary substantially across different systems and applications. Performance characteristics differ markedly between interaction-based and expression-based systems. LOV2-Zdk, cpLID, and iLID demonstrate favorable kinetic profiles in interaction-based systems (Fig. 4d). In expression-based systems, HSF offers the greatest operational simplicity in mammalian systems, whereas YF1 and NmPAL are advantageous in bacterial optogenetic applications (Fig. 4e).

A comparison of representative optogenetic transcription systems reveals distinct strengths and limitations. YF1-FixK demonstrates the lowest background and an exceptional dynamic range (>460-fold) but remains prokaryotic-specific. Moreover, it requires an inverter module to enable light-induced gene upregulation, complicating circuit design and increasing vector payload. EL222 and its variants exhibit low dark-state leakage (<1%) and a moderate dynamic range (8∼ 20-fold) in both bacterial and mammalian systems. However, practical use reveals elevated background and unresolved challenges in nuclear import, calling into question their functional robustness in eukaryotic settings. As an endogenous heat-reponsive factor, HSF offers the simplest architecture and superior compatibility, delivering a 5-fold dynamic range with ∼10% baseline activity, while benefiting from deep-tissue accessibility via thermal stimuli rather than BL. Heterodimerization systems provide low background through constitutive activation but suffer from suboptimal maximum expression (<30%), necessitating targeted optimization for therapeutic relevance.

Collectively, current optogenetic platforms therefore exhibit inherent trade-offs: suppression of basal activity versus maximal output, and prokaryotic tool versatility versus mammalian compatibility. Systematic engineering efforts to resolve these dichotomies are paramount for advancing optogenetic therapeutics toward clinical translation.

### 2.4 Engineering strategies of OTs in translational oncology

**Fig. 5:**
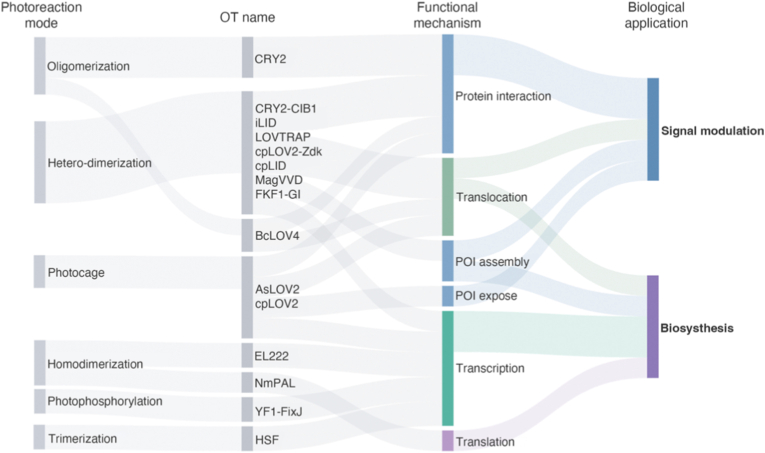
A Sankey diagram mapping the landscape of OTs. The diagram visualizes the connections between major OTs, categorized by their photoreaction mode, functional mechanism, and primary biological application.

The operational principles of OTs are founded on three core mechanisms: light-mediated protein interaction (aggregation/dissociation), photo-induced effector exposure (photocaging), and photo-controlled biosynthesis. These underpin two main therapeutic approaches: optogenetic intracellular signaling modulation and optogenetic biosynthesis (Fig. 5).

#### 2.4.1 Intracellular signaling modulation

Optogenetics enables reversible and precise control of intracellular signaling via several principal mechanisms. Photodimerization systems (e.g., VVD, iLID, cpLID, CRY2/CIB1, and FKF1/GI) enable light-induced homo- or hetero-dimer formation. These processes allow for the assembly of signaling complexes and protein relocalization, thereby modulating key cascades like kinase pathways and receptor activation. A complementary strategy is photocaging, which can be implemented in two ways: controlling the exposure of a POI (e.g., AsLOV2) or releasing a pre-anchored protein from specific organelles using dissociation systems (e.g., LOVTRAP). This versatile approach can be applied to diverse effectors including transcriptional factors, enzymatic regulators (e.g., kinases/phosphatases), trafficking signals, signaling molecules (e.g., G proteins, MAPK), structural proteins (e.g., Rho GTPases), and proteolysis tags (e.g., dTAG/HaloTag), enabling precise regulation of transcription, signal transduction, localization, morphology, and protein stability. Beyond dimerization and uncaging, oligomerization offers another layer of signaling control. Tools such as CRY2 and BcLOV4 undergo light-dependent clustering to amplify or initiate signaling. CRY2PHR, for instance, is particularly effective in activating clustering-dependent cascades including STING, NOD-like receptor, Toll-like receptor, and RTK signaling.

#### 2.4.2 Optogenetic biosynthesis

Optogenetic biosynthesis enables light-controlled expression of virtually any gene, presenting a powerful strategy for cancer immunotherapy through precise modulation of cytokine secretion and therapeutic transgene expression. This approach operates via two main mechanisms: one is transcriptional activation, where light exposure induces the activation or assembly of transcription factors (e.g., YF1-FixJ, EL222-pC120, HSF, p65AD-VVD-Gal4) [109]; and the other is translational regulation, where light modulates mRNA secondary structures to control ribosomal access and protein synthesis, as demonstrated by the NmPAL system [27]. Together, these strategies support programmable, reversible biosynthesis of immunomodulators (e.g., IL-2, IFN-γ, checkpoint inhibitors), improving therapeutic precision and safety.

Although optogenetics has shown exceptional diversity and precision in cancer therapy, its translation to clinical settings is still constrained by limitations in both gene and light delivery. The following section summarizes representative strategies developed to overcome these barriers.

## 3 Optogene and light delivery modalities in cancer therapy

The successful application of optogenetics in oncology critically depends on efficient delivery of optogenetic constructs and activating light to target tissues. Optogene and light delivery constitute two foundational pillars of any optogenetic intervention. Gene delivery governs the spatial precision of optogene expression in tumor or stromal cells, directly shaping therapeutic efficacy and biosafety. Light delivery, in turn, dictates activation depth, tissue selectivity, and real-time controllability, ultimately defining the overall spatiotemporal resolution of the system. Below, we systematically review current strategies for optogene and light delivery, emphasizing their underlying mechanisms, advantages, limitations, and relevance to translational oncology.

### 3.1 Optogene delivery strategies

The efficacy of optogenetic interventions in cancer depends on the efficient delivery of optogenetic constructs, most of which are not natively expressed in mammalian cells. An ideal delivery strategy must fulfill three critical criteria—safety, specificity, and efficiency, while accommodating diverse biological targets ranging from tumor cells to components of the TME (e.g., immune and stromal cells).

#### 3.1.1 Vector selection: *ex vivo* editing vs. *in vivo* delivery

**Fig. 6:**
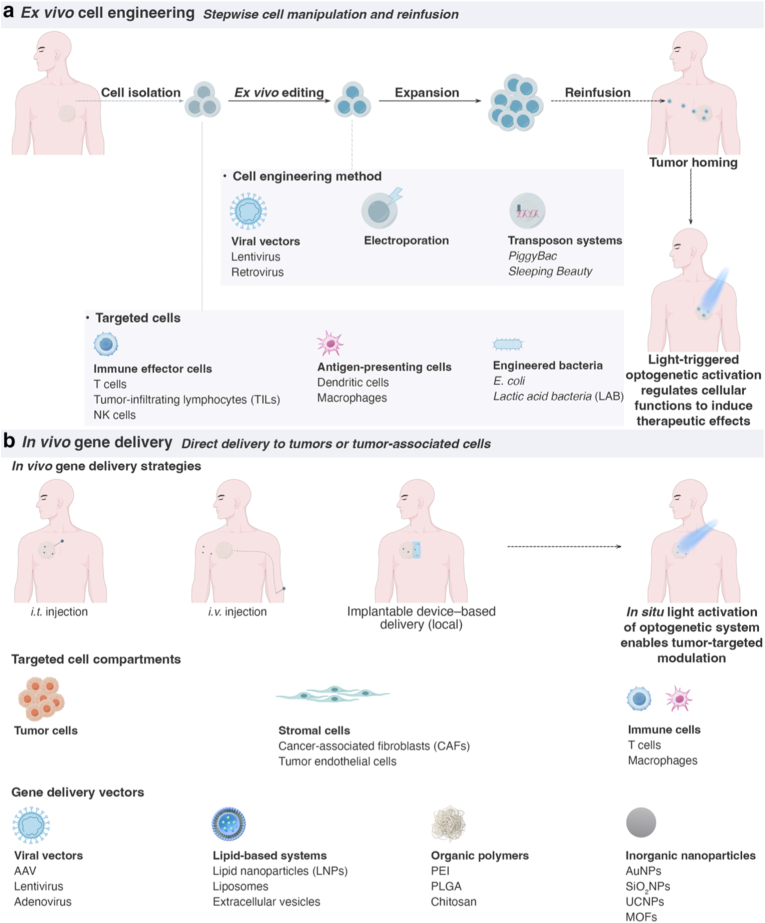
*Ex vivo* and *in vivo* gene delivery strategies for optogenetic cance**r therapy.** (**a**) ***Ex vivo* cell engineering.** Patient-derived cells including immune effector cells, antigen-presenting cells, or engineered bacteria, are isolated, genetically modified using viral vectors, electroporation, or transposon-based systems, followed by *ex vivo* expansion and reinfusion. This strategy enables precise genetic manipulation and quality control prior to administration. Upon reinfusion, engineered cells home to tumor sites. In immune cells, optogenetic modules can be designed to control key effector functions such as CAR activation, cytokine release, or proliferation with high spatiotemporal precision, enabling tunable anti-tumor immunity while minimizing systemic toxicity. In engineered bacteria, optogenetic circuits can regulate localized production of therapeutic payloads (e.g., cytotoxins, immunomodulators) or control bacterial colonization dynamics upon light stimulation. (**b**) ***In vivo* gene delivery.** Gene delivery vectors are administered directly via intratumoral (*i.t.*) or intravenous (*i.v.*) injection, or through implantable local devices, enabling targeted delivery to tumor cells and stromal components within the TME. Multiple vector systems, including viral vectors, lipid-based systems, organic polymers, and inorganic nanoparticles, provide adaptable tools for *in situ* optogenetic modulation. Following delivery, light activation enables optogenetic systems to regulate cellular behaviors within tumors, thereby achieving controlled therapeutic outcomes.

Optogene delivery strategies can be broadly classified into two main approaches: *ex vivo* editing and *in vivo* delivery (Fig. 6). *Ex vivo* approaches involve the isolation of patient-derived cells, such as T cells, mesenchymal stem cells, dendritic cells, macrophages, or tumor-infiltrating lymphocytes, which are then genetically modified *ex vivo*, expanded, and subsequently reinfused into the patient (Fig. 6a). This strategy offers high gene-editing efficiency, allows for the rigorous selection of successfully modified cells, and facilitates the design of personalized therapies. However, *ex vivo* engineering is technically complex, costly, and challenging to scale for solid tumor applications. Furthermore, the *in vivo* persistence of the engineered cells can vary substantially across different cell types. In contrast, *in vivo* delivery involves the direct administration of optogenetic constructs into tumor tissues or target cell populations, either systemically or locally. This strategy simplifies the workflow and is broadly applicable to solid tumors. Nevertheless, this approach faces significant challenges, including limited delivery efficiency, insufficient cell-type specificity, and potential immune responses against the delivery vectors, all of which constrain its translational feasibility.

(1) *Ex vivo* gene editing

For the *ex vivo* introduction of exogenous genes, viral vectors are the most widely adopted platform. Lentiviral and retroviral vectors enable stable genomic integration, supporting long-term transgene expression. Their high transduction efficiency and broad compatibility with dividing cells make them particularly suitable for engineering T cells, NK cells, and stem-cell populations [[110], [111], [112], [113]]. However, permanent genomic integration raises concerns regarding insertional mutagenesis, and large-scale vector production requires specialized facilities and strict regulatory oversight.

As non-viral alternatives, transposon-based systems such as *piggyBac* and *Sleeping Beauty* achieve stable genomic integration through transient expression of a transposase [114,115]. These platforms offer large cargo capacity, high integration efficiency, and reduced manufacturing cost compared with viral vectors, making them attractive for *ex vivo* immune-cell engineering. Transposon systems have been widely applied in CAR-T cell generation and other adoptive cell therapies. Nevertheless, random insertion events and the need for precise control over transposase activity remain important safety considerations.

Electroporation represents the dominant non-viral method for *ex vivo* genome editing [116,117]. By transiently permeabilizing the plasma membrane with short electrical pulses, electroporation enables efficient delivery of plasmid DNA, mRNA, or CRISPR ribonucleoprotein (RNP) complexes. In particular, CRISPR RNP electroporation supports rapid, footprint-free genome modification with minimal long-term genomic perturbation. Despite its versatility, electroporation requires careful optimization to balance editing efficiency, cell viability, and scalability across different cell types.

(2) *In vivo* optogene delivery

**Fig. 7:**
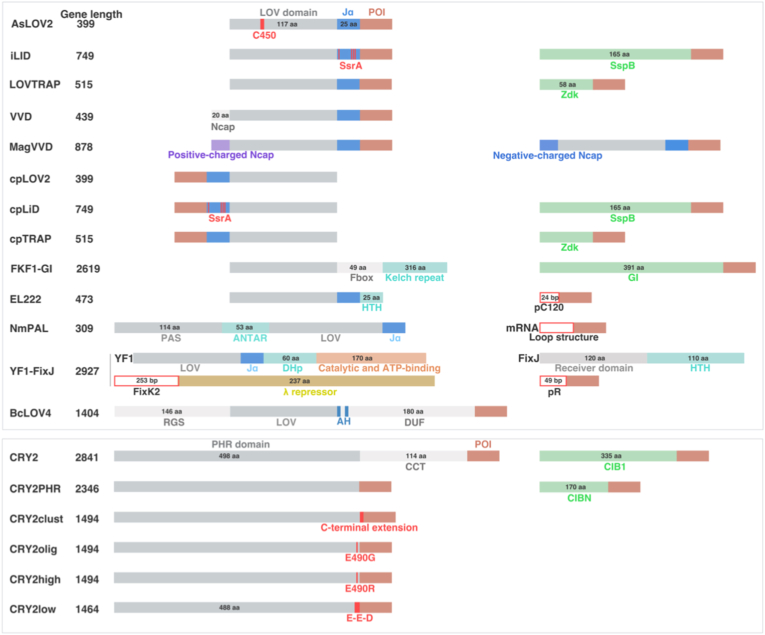
Schematic illustration of the functional domain architecture across various OTs. Each diagram highlights the component domains and their approximate lengths, indicated in amino acids (aa) or base pairs (bp). The reported gene sizes represent the minimal core functional construct, whereas practical implementations often require additional sequences for proper delivery and expression.

In contrast to *ex vivo* gene editing, *in vivo* optogene delivery aims to directly transduce tumor cells and components of TME that cannot be readily isolated, such as tumor-associated macrophages, cancer-associated fibroblasts, and tumor-resident dendritic cells (Fig. 6b). This strategy imposes substantially higher demands on delivery specificity and efficiency, as optogene expression must be restricted to malignant or defined stromal populations to avoid off-target light responsiveness in healthy tissues. Consequently, tumor targeting *in vivo* relies on a combination of delivery route, vector tropism, promoter selection, and microenvironmental cues, rather than post hoc cell selection. Cargo size represents an additional critical constraint for *in vivo* delivery. Many optogenetic systems comprise large coding sequences, particularly non-opsin OTs fused to effectors, regulatory domains, or reporters (Fig. 7). When promoters and regulatory elements are included, these constructs often exceed the packaging limits of certain viral vectors. As transgene size increases, vector production efficiency, genomic stability, and *in vivo* transduction performance generally decline. This limitation makes *in vivo* delivery substantially more challenging than *ex vivo* approaches, where stable integration and clonal expansion can compensate for reduced initial efficiency.

Adeno-associated virus (AAV) is one of the most extensively used viral vectors for *in vivo* gene delivery, owing to its favorable safety profile, low immunogenicity, and capacity for sustained expression in non-dividing cells [22,118]. These properties make AAV attractive for optogenetic applications requiring long-term expression. However, its limited cargo capacity (∼4.7 kb) and lack of intrinsic tumor tropism restrict its use in delivering large or multi-component optogenetic systems. Tumor targeting therefore depends on *i.t.* administration, tumor-selective promoters, or capsid engineering to enhance tumor-specific transduction. As a result, AAV is best suited for localized or regionally confined optogenetic modulation rather than systemic tumor targeting. Adenoviral vectors provide high transduction efficiency, rapid onset of transgene expression, and relatively large packaging capacity, enabling the delivery of complex optogenetic constructs [119,120]. They efficiently infect proliferating tumor cells and are well-suited for *i.t.* delivery. Moreover, conditionally replicative or oncolytic adenoviruses can further enhance tumor selectivity by exploiting cancer-specific replication mechanisms. However, strong innate and adaptive immune responses limit repeated dosing and systemic administration. Consequently, adenoviral vectors are primarily applied via local injection, where tumor selectivity arises from the delivery route and transcriptional control rather than intrinsic specificity. Overall, viral vectors for *in vivo* optogenetic delivery achieve tumor targeting predominantly through local administration, promoter engineering, or capsid modification, rather than through natural tumor tropism.

Non-viral delivery systems offer versatile alternatives to viral vectors, particularly when biosafety, repeatable dosing, or large genetic payloads are required [121]. These platforms use synthetic carriers or biomaterial scaffolds to deliver DNA or mRNA via physicochemical mechanisms, thereby avoiding risks associated with viral integration and immunogenicity. Lipid-based systems are the most established class of non-viral carriers. These formulations typically comprise ionizable or cationic lipids, helper phospholipids, cholesterol, and PEGylated lipids, assemble into liposomes, lipid nanoparticles (LNPs), or lipid–polymer hybrids [[122], [123], [124], [125]]. Their advantages include high encapsulation efficiency, protection of nucleic acids from degradation, scalable manufacturing, and favorable *in vivo* transfection efficiency. However, limitations include limited cell-type specificity, off-target accumulation (notably in the liver), and dose-dependent toxicity. Tumor targeting is commonly achieved through passive accumulation via the enhanced permeability and retention (EPR) effect, ligand conjugation, or stimuli-responsive designs that are sensitive to tumor acidity, enzymatic activity, or redox conditions. Polymer-based delivery systems comprise a diverse group of synthetic and natural macromolecules, including cationic polymers such as polyethyleneimine (PEI) and poly (L-lysine), biodegradable polyesters like poly (lactic-co-glycolic acid) (PLGA), polysaccharides (e.g., chitosan), dendrimers, and block copolymers [[126], [127], [128], [129], [130]]. These carriers offer exceptional structural tunability, controllable degradation, and large cargo capacity, making them attractive for delivering oversized or multifunctional optogenetic constructs. However, polymer-based systems generally exhibit lower transfection efficiency than lipid formulations and may induce cytotoxicity depending on molecular weight and charge density. Tumor targeting relies on ligand conjugation, charge-switching behavior, or microenvironment-responsive release mechanisms.

Inorganic nanomaterials constitute a distinct delivery paradigm based on rigid cores with defined physicochemical properties. Representative materials include gold nanoparticles, mesoporous silica, calcium phosphate, iron oxide nanoparticles, quantum dots, and layered nanostructures [80,110]. These carriers offer high loading capacity, structural stability, and opportunities for multifunctionality, such as imaging guidance, photothermal conversion, or magnetic manipulation. Nevertheless, concerns regarding long-term biodegradability, *in vivo* clearance, and tissue accumulation limit their translational potential. Tumor targeting is typically achieved through surface functionalization, external field guidance, or local administration. Collectively, material-based delivery platforms provide flexible, non-viral solutions for *in vivo* optogenetic gene delivery, particularly for large genetic cargos or multifunctional therapeutic designs. Nevertheless, their tumor selectivity remains largely dependent on engineered targeting strategies and delivery routes rather than intrinsic biological tropism. Therefore, the rational integration of material design with tumor biology and TME characteristics is essential to advance optogenetic cancer therapies toward clinical translation.

### 3.2 Visible light delivery technologies

**Fig. 8:**
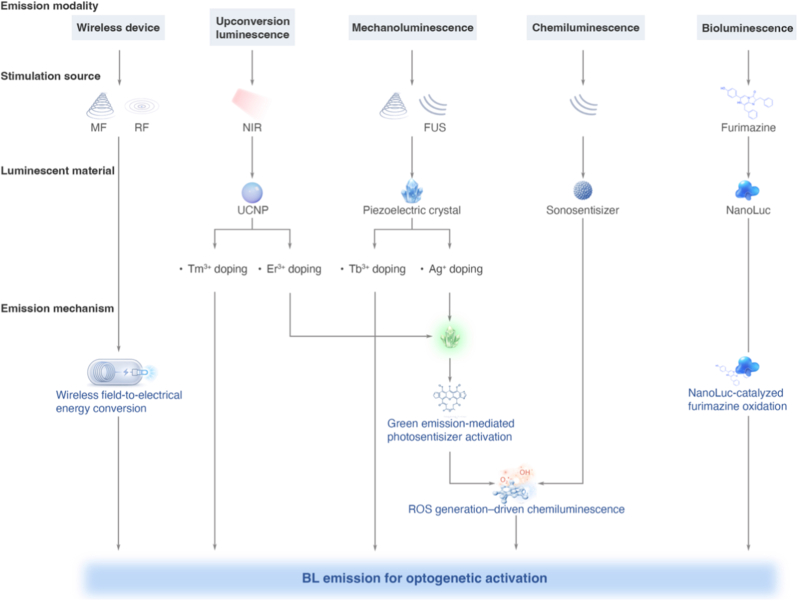
Overview of representative *in vivo* strategies for BL **delivery.** Wireless devices convert externally applied physical fields into electrical energy to power LEDs. Upconversion luminescence utilizes NIR excitation of lanthanide-doped nanoparticles to produce visible emission, which can further activate photosensitizers and induce ROS generation. Mechanoluminescence exploits focused ultrasound–stimulated piezoelectric crystals to generate light via mechanical–electrical energy conversion. Chemiluminescence arises from ROS generated by sonosensitizers or photosensitizer-mediated secondary activation, driving light-emitting chemical reactions. Bioluminescence is achieved through NanoLuc-catalyzed oxidation of furimazine, producing BL via enzymatic emission.

Another major challenge for the clinical application of optogenetic cancer therapies lies in delivering activating light (typically in the visible spectrum) to deep-seated tissues. Although traditional methods such as fiber-optic implantation enable precise illumination, their invasiveness limits both mobility and coverage. The design of non-invasive light delivery systems must therefore navigate several core material and system constraints, including tissue penetration depth, spatial precision, invasiveness, energy conversion efficiency, and biocompatibility. These constraints are often interdependent, and no single strategy simultaneously optimizes all parameters. Consequently, the choice of a suitable light delivery approach is inherently governed by the specific therapeutic context, particularly tumor location, size, and anatomical distribution. To address these challenges, a range of alternative strategies have emerged, including wirelessly powered implantable devices, upconversion luminescence, mechanoluminescence, and bioluminescence (Fig. 8).

#### 3.2.1 Implantable wireless devices

**Fig. 9:**
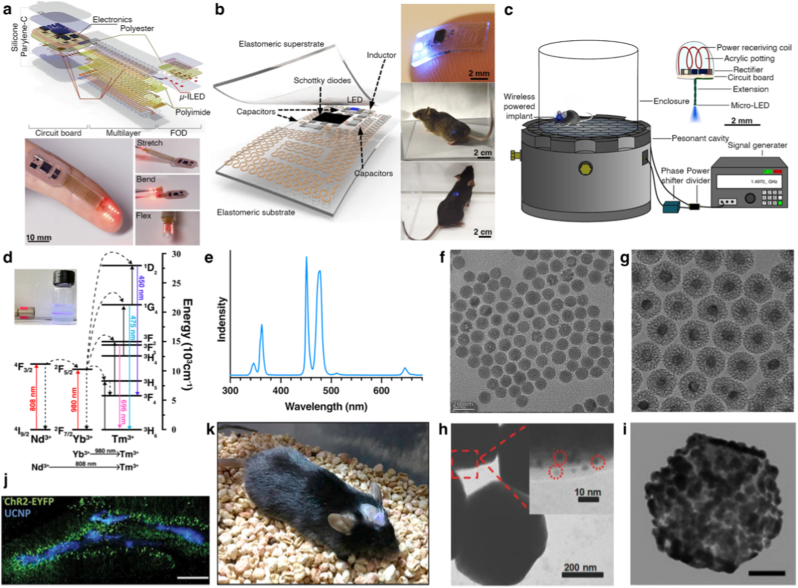
Implantable light-emitting devices and upconversion luminescence platforms for *in vivo* BL **delivery.** (**a-c**) Implantable optoelectronic systems. (**a**) Flexible radiofrequency (RF)-powered light-emitting device for subcutaneous implantation, showing a layered illustration of a wireless optogenetic encoder, the flexible optoelectronic device (FOD) in operation, and controlled illumination patterns of μ-ILEDs during mechanical deformation, demonstrating high flexibility and mechanical robustness. (**b**) Miniaturized RF-powered implantable light-emitting device, illustrating the structural design (elastomeric coil, capacitors, Schottky diodes, and integrated LED) and stable light emission in freely moving animals. (**c**) Magnetically powered light-emitting system, consisting of an external magnetic-field-based power transmitter and an implantable module; the internal coil harvests energy via electromagnetic induction to drive the LED *in vivo*. (**d-k**) UCNPs-based NIR-mediated BL delivery. (**d**) Energy-transfer mechanism of UCNP-based NIR-triggered BL emission, illustrating Nd^3+^/Yb^3+^ sensitization and sequential energy transfer to Tm^3+^. (**e**) Upconversion emission spectrum of Tm^3+^-doped UCNPs under NIR excitation, showing characteristic blue emission peaks. **(f**–**i)** Transmission electron microscopy images of UCNPs with progressive surface modifications: **(f)** NaYF_4_:Yb,Tm core, **(g)** NaYF_4_:Yb,Tm@SiO_2_ core-shell, **(h)** NaYF_4_:Yb,Tm@MOF, and **(i)** NaYF_2_:Yb,Tm@Au. (**j**) Confocal fluorescence image showing colocalization between UCNPs (blue) and ChR2-expressing neurons (EYFP, green) in mouse brain tissue. The blue-light intensity generated by the UCNPs is sufficient to activate ChR2. (**k**) *In vivo* images of animals implanted with a micro-optrode incorporating Tm^3+^-doped UCNPs, demonstrating NIR-triggered deep-tissue BL output. Ref. (**a**) [133], (**b**) [131], (**c**) [132], (**g**) [134], (**h**) [135], (**i**) [136], (**j**) [137], (**k**) [138].

Implantable light-emitting devices represent a promising minimally invasive strategy for optogenetic activation in deep-seated tumors where external illumination is ineffective. These systems typically consist of miniaturized implants integrating microscale light sources (μLEDs), wireless power-harvesting modules, and biocompatible encapsulation layers. Once positioned within or adjacent to tumor tissue, they enable spatially confined and repeatable light delivery without transcutaneous optical fibers, allowing precise control over illumination intensity, timing, and duration while minimizing tissue disruption (Fig. 9a–c) [[131], [132], [133]].

The wireless power transfer is most commonly achieved via radiofrequency (RF) or magnetic-field–based coupling. RF-powered devices convert electromagnetic energy into electrical current through implanted antennas and rectifying circuits, whereas magnetic-field–based approaches (e.g., inductive coupling) offer improved tissue penetration and reduced signal attenuation. Eliminating onboard batteries substantially reduces device size, extends operational lifespan, and permits external, on-demand control of light output. Mechanical flexibility and miniaturization are critical for stable integration within soft, dynamic biological tissues. Flexible designs employing soft substrates, ultrathin electronics, and serpentine interconnects (Fig. 9a and b) minimize mechanical mismatch and improve conformal contact with irregular tumor surfaces. Advances in microfabrication have enabled a transition from centimeter-scale, partially implantable systems (Fig. 9a) to fully implantable millimeter-scale (Fig. 9b), microscale, or even injectable devices, significantly expanding the feasibility of minimally invasive optogenetic interventions [5,[131], [132], [133],^139^,^140^].

Despite these advantages, several limitations remain. Surgical implantation, although less invasive than fiber-based approaches, still poses procedural risks and limits scalability. Miniaturization must balance sufficient light output against thermal management to prevent local overheating. Moreover, illumination is inherently restricted to the vicinity of the implant, complicating the treatment of large or multifocal tumors. Key translational gaps include long-term biocompatibility, mechanical stability under chronic implantation, and robust wireless power coupling *in vivo*, and scalable manufacturing, all of which remain to be addressed for widespread clinical adoption.

#### 3.2.2 UCNP-mediated NIR stimulation

Visible light is strongly attenuated by biological tissues, limiting penetration to <2 mm. In contrast, NIR light (700–1400 nm) penetrates much deeper due to reduced scattering and absorption by endogenous chromophores like hemoglobin and water. Lanthanide-doped upconversion nanoparticles (UCNPs) convert low-energy NIR photons (800–980 nm) into higher-energy ultraviolet or visible emissions (300–800 nm) via excited-state absorption (ESA), matching the activation wavelengths of common OTs (Fig. 9d). This process exploits the unique electronic structure of trivalent lanthanide ions (Ln^3+^). Their 4f orbitals are shielded by outer 5s^2^5p^6^ electrons, making them relatively insensitive to external crystal fields and chemical environments. The energy differences between adjacent electronic states often correspond to energies in the visible and NIR ranges. Upconversion luminescence (UCL) typically involves multiphoton absorption, wherein an ion absorbs multiple low-energy photons to reach a higher excited state. Radiative relaxation back to the ground state then emits a single high-energy photon (Fig. 9d) [141].

UCNPs are synthesized by doping specific proportions of sensitizer and activator ions into matrix materials. The matrix is typically composed of yttrium oxides or fluorides, which offer good transparency, low phonon energy, and thermal stability. Upon NIR absorption, the sensitizer ion reaches an excited state, transferring energy to the activator through non-radiative transitions. The photon energy must align with the energy required for transitions in the sensitizer ion. The most commonly used sensitizers in UCNPs include Yb^3+^, which absorbs 980 nm NIR light to complete the ^2^F_7/2_ → ^2^F_5/2_ transitions, and Nd^3+^, which absorbs 808 nm NIR light for the ^4^I_9/2_ → ^4^F_5/2_ and ^4^F_3/2_ transitions (Fig. 9d and e). Notably, the excited state of Nd^3+^ can transfer energy to Yb^3+^ via non-radiative transitions, thereby exciting the latter. The activator serves as the luminescent core of UCNPs, converting the energy absorbed from the sensitizer into high-energy photons. Activator ions (e.g., Tm^3+^, Er^3+^, Ho^3+^) then emit upconverted light at higher energies. For instance, Tm^3+^-doped UCNPs emit BL (∼450 and 475 nm), matching the activation window of LOV, CRY2, and ChR2 opsins, while Er^3+^-doped UCNPs emit green light (∼520 and 540 nm), and red (∼650 nm) light, suitable for the neuroinhibitory opsins such as Arch.

UCL is a probabilistic process, involving a series of microscopic events (e.g., multiphoton absorption, energy transfer, and electronic transitions) that culminate in low quantum yields for UCNPs. Critical structural determinants of UCNP performance include: (1) Sensitizer and activator concentrations: An optimal balance is crucial, as insufficient doping hinders energy transfer, while excessive doping induces concentration quenching. For NaYF_4_:Yb:Tm nanoparticles emitting BL, the typical molar concentration of Yb^3+^ is around 20%, and Tm^3+^ ranges from 0.5% to 2%. (2) Crystal type and phase: Hexagonal-phase crystals exhibit higher upconversion efficiency compared to cubic-phase crystals due to their better thermal stability, reduced lattice defects, and optimal ion distances. Hexagonal-phase UCNPs are typically synthesized using high-temperature thermal decomposition (280–310 °C), whereas cubic-phase UCNPs are produced under low-temperature hydrothermal methods (180–200 °C) [142]. (3) Particle size: Smaller UCNPs possess a larger surface -to-volume ratio, leading to more surface defects that quench luminescence. Larger particles generally exhibit better efficiency within a certain size range [143]. (4) Surface chemistry: Surface defects or unpassivated active sites can lead to non-radiative relaxation. Similarly, organic ligands can also introduce non-radiative relaxation, causing energy loss. Inorganic shell layers (e.g., NaYF_4_) can passivate surface defects, while a noble metal shell (e.g., Au) can enhance NIR absorption via surface plasmon resonance, thereby improving upconversion efficiency [136]. To further enhance UCL, UCNPs can be integrated with functional materials to form hybrid composites [141]. These strategies can enhance the absorption capability of UCNPs and improve UCL intensity, such as nanometallic particles and organic dyes [144,145], or broaden the absorption spectrum, such as QDs, 2D materials, and metal–organic frameworks (Fig. 9f–i) [[146], [147], [148], [149]].

UCNP-mediated optogenetics has enabled non-invasive neuromodulation and manipulation of the TME [18,58,69,137,150,151]. However, several challenges remain: (1) The limited penetration depth of NIR-I light (700-950 nm) restricts stimulation to superficial tissues (<5 mm). (2) Low quantum yield and significant tissue absorption necessitate high irradiation powers, raising concerns about localized heating. (3) Excitation at 980 nm overlaps with water absorption (950–1050 nm), exacerbating thermal effects. Although 808 nm excitation via Nd^3+^→Yb^3+^→Tm^3+^ reduces heating, it suffers from limited luminous efficiency. (4) Although thermal decomposition yields highly luminescent UCNPs, residual ligands may cause cytotoxicity [152]. Surface modifications, such as PEG, PAA, HA and FA [[153], [154], [155]], or hydrophilic shell coating, such as SiO_2_ [156,157], are essential to enhance the safety of UCNPs. However, these may paradoxically introduce quenching defects, reabsorption, or non-radiative losses. Key translational gaps include scalable synthesis with batch-to-batch consistency, comprehensive long-term toxicity profiling, and integration with clinically approved delivery vehicles.

#### 3.2.3 Magnetoluminescence

Although UCL provides high spatiotemporal specificity, the intensity of visible light generated under NIR excitation is often insufficient to insufficient activate OTs in deep-seated tumors (>3 cm). Mechanoluminescence-based strategies offer a compelling alternative by exploiting deeply penetrating physical stimuli—such as alternating magnetic fields (MF) or focused ultrasound (FUS)—to generate light *in situ*, thereby enabling volumetric illumination beyond the reach of conventional photonic approaches [158,159].

Mechanoluminescence refers to light emission induced by mechanical stimulation of luminescent materials. In biomedical contexts, mechanical energy can be delivered non-invasively through MF or FUS, both of which penetrate deep tissues with minimal attenuation. The mechanical deformation of mechanoluminescent materials is transduced into electronic excitation, ultimately yielding visible photon emission. By decoupling light generation from direct optical excitation, mechanoluminescence enables internal illumination at depths inaccessible to external light delivery, making it particularly attractive for deep-tissue optogenetics.

**Fig. 10:**
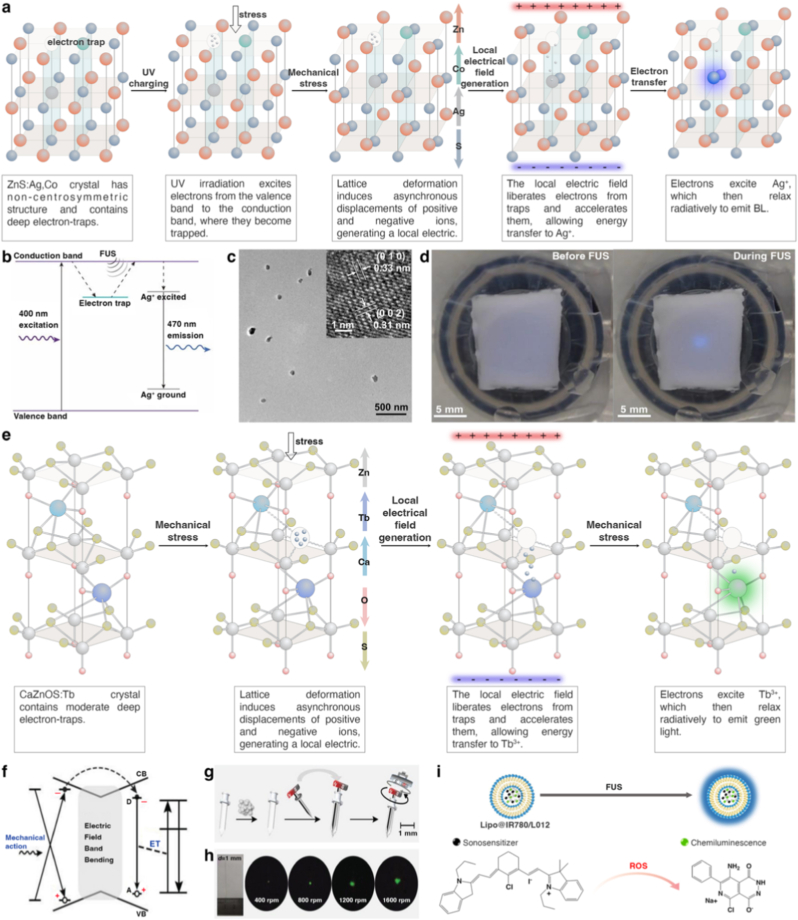
Piezoelectric crystals-based mechanoluminescence and sonoluminescence for *in vivo* BL **delivery.** (**a, b**) Mechanoluminescence mechanism of ZnS:Co,Ag crystal. Co^2+^ doping introduces deep electron traps within the ZnS lattice. Upon UV irradiation, electrons are excited from the valence band to the conduction band and subsequently captured by these deep traps. During the piezoelectric process, mechanical deformation induced by FUS or a magnetic field generates a local electric field that releases and accelerates the trapped electrons. These high-energy electrons excite Ag^+^ ions, resulting in 470 nm light emission. (**c**) TEM image and high-resolution TEM image (insert) of Zns:Ag,Co nanoparticles. (**d**) Photographs of a PDMS phantom placed at the focus of the ultrasound transducer, exhibiting minimal emission prior to FUS stimulation and distinct blue mechanoluminescence during FUS application. (**e, f**) Green mechanoluminescence mechanism of Tb^3+^-doped CaZnOS. Tb^3+^ doping introduces moderate-depth electron traps within the CaZnOS lattice. Mechanical stress induces asymmetric ionic displacements, generating a local piezoelectric field that liberates and accelerates trapped electrons. These electrons excite Tb^3+^ ions, which undergo radiative relaxation to yield green mechanoluminescence. (**g**) Schematic of an implantable magneto-luminescence microdevice device and (**h**) light intensity output at different magnetic rotation frequencies. (**i**) Mechanism of FUS-triggered sonoluminescence from Lipo@IR780/L012 nanoparticles. FUS activates the sonosensitizer IR780 to generate reactive oxygen species (ROS). The ROS subsequently oxidize L012, triggering chemiluminescence that produces blue emission. Ref. (**b-d**) [160], (**f-h**) [159], (**i**) [161].

Among mechanoluminescent systems, piezoelectric materials have emerged as a central platform for their ability to convert mechanical stress into localized electric fields. Deformation of non-centrosymmetric piezoelectric lattices induces charge separation and transient internal electric potentials, which can liberate carriers trapped in defect states and accelerate free electrons. Subsequent energy transfer to luminescent dopants results in light emission, with efficiency and emission spectra governed by trap depth, defect density, and dopant identity [158,159]. For instance, BL emission can be achieved in piezoelectric ZnS nanocrystals co-doped with Co^2+^ and Ag^+^. In this system, Co^2+^ doping introduces deep electron traps within the bandgap for efficient charge storage [160]. Upon pre-irradiation with ultraviolet (UV) light, electrons are excited from the valence band to the conduction band and subsequently captured by these deep traps. Mechanical stimulation then releases and accelerates trapped electrons via piezoelectric fields, enabling excitation of Ag^+^ centers and BL emission suitable for optogenetic activation (Fig. 10a–d) [160].

Current limitations include: (1) light output is often insufficient for efficient optogenetic activation, requiring further material optimization; (2) reliance on UV pre-charging complicates *in vivo* use and limits long-term or repeated stimulation; (3) device configurations, material formulations, and stimulation protocols remain at the proof-of-concept stage. Key translational gaps include improving quantum efficiency, eliminating the need for pre-charging, establishing safety profiles for implanted materials, and developing clinically compatible stimulation devices.

#### 3.2.4 Chemiluminescence

Chemiluminescence exploits tumor-associated biochemical features, particularly elevated reactive oxygen species (ROS), to achieve spatially confined light generation *in vivo*. A representative system employs horseradish peroxidase (HRP) to catalyze the oxidation of luminol in the presence of H_2_O_2_, producing BL (∼425 nm) via a radical-mediated reaction cascade [48]. When HRP-encoding genes are co-delivered with optogenetic constructs, this approach simplifies delivery requirements, as only the small-molecule substrate (luminol) requires systemic administration. Spatial specificity is inherently enhanced by the ROS-enriched TME, which confines light emission predominantly to malignant tissues. Importantly, chemiluminescent activation is reversible and tunable: depletion of H_2_O_2_ or luminol rapidly extinguishes light emission, whereas substrate replenishment restores activity. Compared with inorganic nanoparticle-based light sources, chemiluminescence offers superior biocompatibility and metabolic clearance. Nonetheless, rapid substrate consumption and limited photon flux pose challenges for sustained optogenetic activation, motivating the development of signal-amplifying hybrid strategies.

One emerging solution involves coupling chemiluminescence with mechanoluminescence to boost ROS availability and light output within the TME. In such hybrid systems, piezoelectric materials (e.g., Tb-doped CaZnOS nanocrystals) act as mechanically driven ROS amplifiers. Under mechanical stimulation, liberated charge carriers excite Tb^3+^ ions, generating green emission that activates photosensitizers to produce ROS. These ROS subsequently fuel chemiluminescent reactions, yielding secondary BL emission. Although mechanistically more complex, this cascade circumvents the need for UV pre-charging and improves biological compatibility (Fig. 10e–h) [159].

Chemiluminescence is uniquely suited for tumors with elevated endogenous ROS, such as inflammatory or hypoxic tumors. It does not require external irradiation, making it applicable to tumors at virtually any depth, provided that the enzymatic components and substrates can be delivered. However, its applicability is limited in tumors with low basal ROS levels, where insufficient light output may preclude effective optogenetic activation.

Limitations include: (1) sustained activation requires repeated substrate administration; (2) light output is generally low compared to device-based approaches; (3) ROS levels vary significantly across tumor types and stages, making efficacy unpredictable. Key translational gaps include improving substrate pharmacokinetics, developing signal-amplifying strategies, and validating performance across diverse tumor models with varying ROS profiles.

#### 3.2.5 Bioluminescence

Bioluminescence provides an intrinsically biocompatible strategy for deep-tissue light delivery through engineered luciferase systems. NanoLuc, an ATP-independent luciferase, catalyzes the oxidation of furimazine (Fz) to emit BL at 460 nm. Recent advancements in substrate engineering have addressed limitations of early-generation compounds. For instance, the redesigned substrate fluorofurimazine (FFz) exhibits superior brightness (>10-fold increase), enhanced aqueous solubility, and improved pharmacokinetic properties compared to Fz, while maintaining low systemic toxicity in preclinical models [69,162]. NanoLuc can be delivered via *i.t.* injection or encoded by optogenetic vectors, while its substrate is administered systemically. Light emission occurs locally upon substrate diffusion into the tumor, minimizing off-target effects. This approach is compatible with both prokaryotic and eukaryotic optogenetic systems.

Bioluminescence enables activation in disseminated or systemic tumors, provided that luciferase expression is selectively targeted to malignant cells. It is particularly well-suited for metastatic disease where multiple tumor sites preclude local device implantation or focused external stimulation. However, it is less suitable for tumors requiring high-intensity or rapidly switchable activation due to relatively low light output and substrate-dependent kinetics.

Limitations include: (1) although improved substrates enhance brightness, light emission intensity remains lower than device-based approaches; (2) rapid substrate clearance may require repeated dosing; (3) during cell division, luciferase expression may diminish over time. Key translational gaps include optimizing substrate half-life, achieving stable and selective luciferase expression in target cells, and validating scalability for systemic tumor burdens.

Collectively, these non-invasive light delivery strategies are governed by distinct design trade-offs that arise from the coupling between light generation mechanisms and delivery modalities (Table 2). Their applicability to different tumor types is determined not only by optical penetration, but also—more fundamentally—by the spatial precision of light source localization, which in most cases depends on the delivery and retention of light-emitting materials or genetically encoded components.

**Table 2:** Comparative analysis of non-invasive light delivery strategies for optogenetic cancer therapy.

Parameters	Implantable devices	Upconversion luminescence	Mechanoluminescence	Chemiluminescence	Bioluminescence
Light intensity	High	Medium-low	Low-medium	Low	Low
Penetration depth	Deep (localized)	Moderate deep	Deep	Local	Moderate (diffuse)
Spatial precision	High	High	Medium-Low	Low-Medium	Low
Temporal precision	High	High	High	Low	Low
Invasiveness	High	Low	Low	Low	Low
Controllability	High (real-time, programmable)	Medium (dependent on external NIR)	Low (limited temporal control)	Low (irreversible or limited control)	Medium (substrate-dependent activation)
Tumor suitability	Localized solid tumors; well-defined boundaries	Superficial to moderate depth (<5 mm); targetable via passive/active delivery	Deep-seated (>3 cm); volumetric illumination possible	ROS-enriched tumors; depth-independent	Disseminated/metastatic; systemic disease
Key limitations	Invasiveness, fixed coverage, surgical risks	Limited NIR penetration, low efficiency, 980 nm heating, cytotoxicity concerns	Low emission, UV pre-charging, immature integration	Rapid substrate consumption, low photo flux, tumor-dependent efficacy	Low absolute output, substrate clearance, enzyme dilution over time
Translational gaps	Long-term biocompatibility, scalable manufacturing	Batch consistency, toxicity profiling, clinical integration	Efficiency improvement, elimination of pre-charging, safety validation	Substrate optimization, signal amplification, validation across tumor types	Optimized substrate half-life, stable expression, scalability

From a physical perspective, different strategies exhibit inherent trade-offs between penetration depth and light generation efficiency. Implantable devices and externally powered systems driven by RF or magnetic fields provide superior penetration and controllability, positioning them closer to clinical translation. Mechanoluminescent approaches, activated by ultrasound or magnetic stimulation, also benefit from high tissue penetration but are currently limited by low emission intensity and immature system integration. Upconversion systems enable wavelength conversion with relatively precise spectral control but face constraints in NIR penetration depth and thermal management. Bioluminescent and chemiluminescent systems bypass depth limitations altogether but introduce new constraints related to substrate delivery, enzymatic stability, and tumor biochemistry.

Overall, the selection of an appropriate light delivery strategy should be guided by an integrated consideration of tumor characteristics (depth, size, anatomical distribution, ROS profile, and disease burden), delivery efficiency, and activation modality. Despite substantial progress, a significant gap remains between current experimental systems and clinical implementation, particularly in terms of scalable manufacturing, long-term biosafety, precise dose control, and the integration of light delivery with optogene expression systems. Addressing these interconnected challenges will be essential for advancing optogenetic cancer therapies toward clinical translation.

## 4 Therapeutic applications in oncology: from mechanistic control to clinical translation

With the continued diversification of OTs and the rapid expansion of preclinical studies, an increasing number of optogenetic strategies have been validated in tumor models. These approaches achieve therapeutic effects through light-dependent, programmable control of intracellular signaling and transgene expression with high spatiotemporal precision. Representative applications include gene editing, CAR-T cell activation, immune modulation, induction of apoptosis, and prevention of postoperative recurrence (Table 3). In the following sections, we first summarize established applications based on well-characterized optogenetic systems, and then discuss emerging platforms with distinct activation properties as complementary extensions of the optogenetic toolkit. Finally, we highlight combination strategies that integrate optogenetics with conventional therapies to enhance translational potential.

**Table 3:** Translational applications of optogenetic cancer therapy.

Tumor model	Target cells	OTs	Optogene delivery approach	Mechanism of action	Light delivery approach	Optical parameters	*In vivo* efficacy	Ref.
Melanoma (B16-OVA) Lewis lung carcinoma (LL/2)	BMDCs	CRY2clust	*In situ* injection	STING activation	Bule LED	470 nm, 2 mW/cm^2^, 30 min ON/OFF cycle for 6 h each day	∼45% tumor reduction (vs anti-PD-1); additional ∼50% reduction with combination therapy	[163]
Melanoma, Lymphoma (Raji)	CD8^+^ T cells	iLID CRY2-CIBN	Adoptive transfer of LiCAR T cells (UCNPs modified)	CAR-T cell activation	UCNPs-mediated NIR	980 nm, 250 mW/cm^2^, 20 s ON + 5 min OFF for 2 h each day	∼40% reduction in tumor volume	[18]
Cervical cancer	Hela	CRY2-CIB1	*i.t.* injection of UCNPs@PEI@plasmid	Apoptosis	UCNPs-mediated NIR	980 nm, 4 W, 10 s ON +50 s OFF for 2 h each day	∼80% reduction in tumor volume	[164]
Breast/liver cancer	*E. coli*	EL222	IV injection of *E.coli*@UCNPs	Immunol activation of CD8^+^ T cell and direct tumor killing	UCNPs-mediated NIR	808 nm, 0.6 W/cm^2^ 10 min each day	∼85% reduction in tumor volume	[165]
A549 lung adenocarcinoma	A549	HSF	-	Photothermal-mediated CRISPR-Cas9 activation	Photothermal (NIR-II)	1064 nm, 0.33W/cm^2^, discontinuous irradiation for 30 min (maintain 41.5 °C)	near-complete tumor growth inhibition	[80]
Cervical cancer	Hela	CRY2	*i.t.* injection of UCNPs	RIPK1-RIPK3-TNFR mediated necroptosis	UCNPs-mediated NIR; NanoLuc-mediated bioluminescence	980 nm, 250 mW/cm^2^, 20 s ON + 5 min OFF for 2 h every three days	near-complete tumor growth inhibition	[69]
Lymphoma	CD8^+^ T cells	cpLOV2	*i.t.* injection of CAR-T cells	CAR-T cell activation	UCNPs-mediated NIR illumination	980 nm, 30 mW/mm^2^ 1 min ON/OFF ^for^ 4 or 8 h each day	suppressed postoperative tumor recurrence	[166]
Y79 retinoblastoma	Y79	CRY2-CIB1	*i.t.* injection of NanoLuc and plasmid	FADD-FAS mediated caspase-3 activation and apoptosis	*i.p* injection of fluorogenic substrate	-	∼70-fold inhibition of tumor growth	[167]
4T1 breast cancer	4T1	HSF	-	Photothermal-mediated IFN-γ expression triggers TAM repolarization	Photothermal material	808 nm, 0.5W/cm^2^, 30 min	-	[78]
CT26 colon cancer	*E. coli*	YF1-cI	IV injection of *E.coli*@UCNPs	BL-induced TRAIL expression in *E. coli* triggers tumor cell death	UCNPs-mediated NIR	-	∼5-fold increase in tumor growth inhibition	[168]
B16F10 melanoma	*Lactococcus lactis*	YF1-cI	Oral gavage	BL-induced IFN-γexpression in *E. coli* activates immunity	UCNPs-mediated NIR	808 nm, 1W/cm^2^, 5 min for 3 repeats	near-complete tumor ablation	[169]
H1299, HepG2	Oncolytic adenovirus	VVD	*i.t.* injection	BL-mediated p65-VVD-Gal4 homodimerization induces E1 expression	Implantable blue LED	470 nm, 90 mW/cm^2^, 8 h/day for 10 days	near-complete tumor ablation	[109]
A20 lymphoma	*Salmonella* (ΔXIV)	PadC	*i.t.* injection	NIR-mediated transcription of antitumor proteins	Direct NIR	710 nm, 10 mW/cm^2^, 2 h/day for 10 days	near-complete tumor growth inhibition	[170]
Breast cancer	-	VChR1	*i.t.* injection of Zn-ZIF@UCNP@VChR1	ZIF releases metal cations; AUCNP provides upconverted green light for activation	UCNPs-mediated NIR	980 nm, 1 W/cm^2^ for 20 min	∼5-fold increase in tumor growth inhibition	[171]
Breast cancer	MCF-7	HSP70	-	-	Carbon nanotube-mediated NIR	1.5 W/cm^2^ for 1 min	∼3-fold increase in tumor growth inhibition	[172]
Hela cervical cancer	Hela	CRY2-CIB1	-	OS-mediated p53 translocation induces autophagy	UCNPs-mediated NIR	980 nm, 2 mW/mm^2^, 2 min ON and 1min OFF for 4 h	-	[92]
B-lymphoblastic leukemia	Human T cells	CRY2-CIB1 ASLOV2	*i.t.* injection of edited T cells	OS-mediated transcription factor assembly and CAR expression	*Ex vivo* blue LED	370 nm, 5 mW/cm^2^, 1 s ON + 30 s OFF for 24 h	near-complete tumor growth inhibition	[74]

### 4.1 Established optogenetic applications based on well-characterized systems

**Fig. 11:**
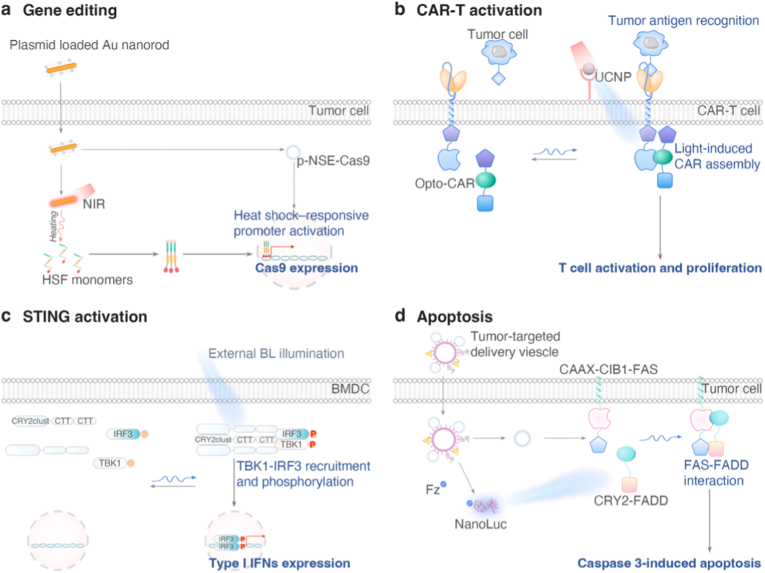
*In vivo* optogenetic strategies for tumo**r therapy.** Schematic overview of representative *in vivo* applications of OTs for cancer treatment. (**a**) **Optogenetic gene editing.** Plasmids encoding sgRNA and Cas9 are delivered into tumor cells through Au-based gene delivery vectors. Upon external NIR irradiation, the Au nanorods generate localized heat that induces trimerization of endogenous HSF. The activated HSF binds to HSE on the plasmid, initiating Cas9 transcription and enabling light-controlled gene editing within tumor cells. (**b**) **Optogenetic CAR-T cell activation.** In this system, the chimeric antigen receptor (CAR) is spited into two fragments, each fused to one component the optogenetic heterodimerization pair LOV2–SsrA and SspB. Following tumor antigen recognition and NIR stimulation, UCNP-modified LiCAR T cells convert NIR to blue emission, inducing LOV2–SsrA/SspB dimerization. light-triggered reconstitution of the complete CAR leads to T cell activation and enhanced tumor-cell killing. (**c**) **Optogenetic STING activation.** The critical C-terminal tail (CTT) domain of STING is fused to the highly photosensitive CRY2clust module. External BL illumination is sensed by *in vivo* CRY2clust, which drives oligomerization of the attached CTT domain. This light-induced assembly activates the STING pathway and promotes downstream innate immune signaling. (**d**) **Optogenetic induction of apoptosis in tumor cells.** A folate-targeted carrier delivers an optogene together with NanoLuc into retinoblastoma cells. Following systemic administration of the NanoLuc substrate furimazine (Fz), the intracellular NanoLuc generates blue bioluminescence that activates an optogenetic module designed to promote FAS–FADD dimerization. Light-induced assembly of FAS and FADD triggers downstream caspase activation, ultimately leading to tumor-cell death.

While gene editing technologies such as CRISPR-Cas9 hold immense promise for modulating tumor cell behavior, systemic delivery of edited tools raises concerns over off-target effects and unintended genomic modifications. Optogenetics address this by enabling light-inducible activation, thereby restricting editing activity to illuminated regions. For instance, a photothermal CRISPR system operating in the NIR-II window (1000–1350 nm) was developed to facilitate deep-tissue genome editing [80]. This system employed cationic polymer-coated gold nanorods as photothermal transducers, converting NIR-II light into localized heat. This triggered the trimerization of HSF, which bound to HSE promoters to drive Cas9 expression, while sgRNA was constitutively expressed via a U6 promoter. This strategy enabled targeted genome disruption in subcutaneous A549 tumors with reduced off-target effects, illustrating how optogenetics combined with photothermal nanomaterials can improve precision and tissue penetration (Fig. 11a).

Immunotherapies, including CAR-T cell therapies, are often limited by systemic toxicities such as cytokine release syndrome and on-target/off-tumor effects [18]. Optogenetics offers spatiotemporal control over immune activation, helping to mitigate these risks. Most optogenetic immunotherapies involve *ex vivo* gene editing of immune cells (e.g., T cells, NK cells, macrophages) or bacteria with light-responsive circuits. These engineered cells are adoptively transferred and localized to tumor sites via active or passive targeting, where light illumination triggers localized immune activity.

A prominent example is the light-switchable CAR (LiCAR) T cell system [25]. Conventional CARs consist of an extracellular single-chain variable fragment (scFv), a transmembrane domain, and intracellular signaling modules (CD3ζ and co-stimulatory domains). Antigen binding induces conformational changes that expose ITAM motifs within CD3ζ, initiating phosphorylation, kinase recruitment, and downstream signaling that culminates in T-cell activation. The LiCAR system splits the CAR into two components: a membrane-anchored antigen-binding module (scFv-TM-4-1BB) and a cytoplasmic signaling module (CD3ζ-4-1BB), each fused to optogenetic dimer (CRY2/CIBN or LOV2-SsrA/SspB). BL induces dimerization, reassembling the functional CAR and triggering T-cell activation and tumor killing. When coupled with UCNPs for NIR-to-BL conversion, this system enables precise activation in deep tissues with reduced systemic toxicity (Fig. 11b).

The stimulator of interferon genes (STING) pathway, a key mediator of antitumor immunity, is another target for optogenetic modulation. Conventional STING agonists often cause systemic inflammation. To overcome this, *Dou* et al. engineered a CRY2-based optogenetic platform to spatially control the STING activation. By fusing repeated STING CTT domains (the key functional domain of STING) to CRY2clust (with ultra-light-sensitivity to enable external BL stimulation), BL-induced CTT oligomerization recruited TBK1 and IRF3, driving localized interferon production. This strategy enhanced dendritic cell (DC) maturation and cytotoxic T-cell responses in melanoma models, demonstrating reduced off-tissue immunotoxicity (Fig. 11c) [163].

Direct modulation of intracellular signaling to induce apoptosis, necroptosis, and bystander cytotoxic effects is another promising optogenetic strategy. For example, Ding et al. developed a bioluminescence-driven optogenetic system for retinoblastoma therapy [167]. They constructed folate acid (FA)-modified vesicles co-delivering NanoLuc luciferase and optogene encoding membrane-anchored FAS-CIB1 and cytoplasmic CRY2-FADD. Following *i.v.* administration of the luciferase substrate (Nano-Glo), localized bioluminescence activated CRY2-CIB1 dimerization, driving FAS–FADD complex assembly and subsequent caspase-3–dependent apoptosis (Fig. 11d). Besides, Lian et al. designed an optogenetic necroptosis system by fusing RIPK3 to CRY2 [60]. BL-induced oligomerization of CRY2-RIPK3 activated MLKL phosphorylation, leading to rapid plasma membrane disruption and a pro-inflammatory cell death. This approach achieved tumor ablation in subcutaneous HeLa models using both UCNP-mediated NIR irradiation and bioluminescent activation, highlighting the capacity of optogenetic systems to elicit bystander killing in heterogeneous tumor environments [69].

### 4.2 Expanding the optogenetic toolkit: red- and far-red-responsive systems

In addition to the extensively developed LOV-, CRY2-, and HSF-based BL-responsive systems, a number of optogenetic platforms responsive to red or far-red light (FRL) have also been developed. These systems offer a distinct advantage in tissue penetration due to the longer activation wavelengths, making them particularly relevant for treating deep-seated or poorly accessible tumors. However, compared with well-characterized blue-light counterparts, red/FRL systems generally exhibit lower tool diversity, more complex photochemistry, and requirement of exogenous chromophore supplementation in many cases. Consequently, their application in oncology remains at an earlier stage of development. This section provides a systematic overview of representative platforms, highlighting their molecular mechanisms, translational potential, and current limitations. A broader comparative summary is provided in Table 1.

#### 4.2.1 PhyB-PIF

The plant-derived PhyB–PIF module is a light-inducible heterodimerization system that enables reversible control of protein interactions. Red light illumination (∼630–656 nm) induces the association between PhyB and PIF, whereas FRL (∼740–780 nm) triggers their dissociation, allowing bidirectional and dynamic regulation. Based on this mechanism, Morgane Haeger et al. engineered a light-inducible T cell activation system by fusing PIF6 to a TCR-targeting Fab fragment [173]. Upon red light exposure, PIF6–Fab interacts with immobilized PhyB, inducing clustering of TCR complexes and triggering downstream T cell activation.

Although relatively few studies have directly applied PhyB–PIF systems in preclinical cancer models, this platform has been widely utilized for regulating transgene expression, transposon activity, and intracellular signaling. These capabilities suggest substantial potential for oncological applications requiring precise and reversible control. However, the system presents several notable limitations: the relatively large size of PhyB (∼755 aa) complicates viral packaging, and the requirement for the exogenous chromophore PCB (absent in mammalian cells) restricts *in vivo* use, often limiting applications to *ex vivo* engineered systems.

#### 4.2.2 BphP1/Q-PAS1

Bacteriophytochrome-based systems, such as the BphP1–QPAS1 module, represent an alternative class of NIR-responsive optogenetic heterodimerization tools derived from bacterial photoreceptors. Activated by far-red or NIR light (∼740–780 nm), these systems enable deeper tissue penetration. Notably, they utilize biliverdin as a chromophore, an endogenous metabolite in mammalian cells, eliminating the need for exogenous supplementation.

The BphP1–QPAS1 system has been widely applied for light-controlled transcriptional regulation, typically through fusion with transcriptional activators (e.g., VP16) and DNA-binding domains (e.g., Gal4) [174,175]. Despite these advantages, several limitations remain: the large size of BphP1 increases viral packaging difficulty, and compared with well-established BL systems, its dynamic range and switching kinetics are generally more limited.

#### 4.2.3 BphS

BphS is a bacteriophytochrome-derived FRL-responsive optogenetic enzyme that functions as a light-activated diguanylate cyclase. Upon FRL illumination, BphS catalyzes the conversion of intracellular GTP into cyclic di-GMP (c-di-GMP), which subsequently induces homodimerization of a c-di-GMP–responsive transcriptional activator (e.g., p65–VP64–BldD), driving transgene expression. Based on this mechanism, Yu et al. developed a hydrogel-based platform for the prevention of postoperative tumor recurrence [166]. Engineered cells expressing the BphS system were encapsulated within an implantable hydrogel placed at the surgical site. Upon FRL illumination, the system enabled controlled expression of immunostimulatory cytokines (IFN-β, TNF-α, and IL-12), significantly reducing melanoma recurrence.

As an enzymatic amplification system, BphS enables signal transduction through second messenger accumulation, allowing robust transcriptional activation even under low light intensities. However, its multicomponent nature increases system complexity, and precise control of c-di-GMP levels requires careful optimization.

A range of additional red- and FRL-responsive optogenetic systems, including PadC [170], Cph1 [176], DrBphP [177], and PhyA/FHY1 [178], have been developed with diverse regulatory mechanisms. Although these platforms are less extensively characterized than BL-responsive systems, their long-wavelength activation properties confer unique advantages for deep-tissue and *in vivo* applications. Accordingly, despite current limitations in tool diversity and engineering maturity, red/far-red optogenetic systems represent an important and promising direction for future development in optogenetic cancer therapy.

### 4.3 Combination strategies integrating optogenetics with conventional cancer therapies

Despite the precise spatiotemporal control offered by optogenetic systems, the complexity and heterogeneity of tumors often limit the efficacy of monotherapies. Integrating optogenetics with established therapeutic modalities therefore represents a promising strategy to enhance treatment outcomes while minimizing systemic toxicity. For example, Zhou et al. developed an optogenetically activated STING signaling system in DCs for the treatment of LL/2 tumors. This approach demonstrated a significantly enhanced tumor-suppressive effect compared with conventional anti-PD-L1 therapy alone, resulting in an approximately 45% reduction in tumor volume. Notably, when combined with anti-PD-L1 treatment, optogenetic STING activation further improved therapeutic efficacy, yielding an additional ∼50% decrease in tumor volume compared with optogenetic STING activation alone [179].

In addition to immunotherapy, optogenetic strategies can also be effectively combined with conventional chemotherapeutic agents [180]. For example, optogenetic regulation of macrophage polarization using the LOV2–STIM1 system enables sustained M1 activation under light stimulation, thereby enhancing antitumor immune responses. When combined with temozolomide, this approach results in synergistic tumor inhibition in melanoma models, resulting in an additional ∼50% decrease in tumor volume. Mechanistically, this synergy is associated with remodeling of the tumor immune microenvironment, including reversal of chemoresistance and increased infiltration of cytotoxic CD8^+^ T cells. These findings highlight the potential of optogenetics to improve chemosensitivity and augment the efficacy of existing chemotherapy regimens [181].

Besides, optogenetics can also be integrated with surgical interventions. Surgical resection remains a first-line treatment for many solid tumors; however, incomplete removal and residual tumor cells often lead to postoperative recurrence. In this context, optogenetic strategies offer a unique opportunity for adjuvant therapy by enabling precise, localized modulation of the tumor microenvironment at the surgical site. Optogenetic transcriptional systems are particularly well suited for this scenario, as they enable sustained, light-controlled expression of therapeutic factors. For instance, optogenetically engineered cells or bacteria can be encapsulated together with light-delivery materials within biocompatible hydrogels and implanted into the resection cavity. This approach allows localized and prolonged therapeutic activation while also facilitating retention of light-responsive materials and potential removal after treatment. Importantly, while such strategies are commonly implemented with BL-responsive systems, red- and FRL-responsive optogenetic platforms offer additional advantages by enabling deeper tissue penetration and, in certain cases, direct external illumination without the need for implanted light-conversion materials, particularly for superficial tumors [166].

Overall, the high spatiotemporal precision of optogenetic systems provides a strong foundation for their integration with conventional cancer therapies. Through such combinatorial strategies, optogenetics can enhance therapeutic efficacy while reducing the required dosage of cytotoxic agents, thereby mitigating treatment-related side effects. In addition, optogenetic approaches can be further integrated with gene delivery systems or light-responsive biomaterials to enable multimodal therapies, including photodynamic and photothermal treatments [168,169,182,183]. Although current reports on such combinatorial strategies remain limited, their intrinsic programmability and precise controllability highlight substantial potential for synergistic therapeutic applications. Continued development in this area is expected to further expand the scope and effectiveness of optogenetic cancer therapy.

## 5 Challenges and translational roadmap

Despite rapid advances in optogenetic technologies, their clinical translation has thus far been largely confined to opsin-based systems for neurological and ophthalmological indications. Translating optogenetic strategies into effective cancer therapies faces several fundamental challenges. These include the need for context-specific optimization of optogenetic components tailored to distinct tool architectures and tumor types, the development of efficient and targeted gene delivery strategies, and the establishment of safe and effective light delivery approaches within the complex TME. A comparative overview of key translational parameters across representative optogenetic systems is provided in Table 4, highlighting the diverse molecular mechanisms, spectral properties, cofactor requirements, and host compatibility that collectively influence their suitability for *in vivo* oncology applications.

**Table 4:** Comparative summary of key translational parameters of representative optogenetic systems.

Category	Regulation mechanism	Activation wavelength	Tissue penetration	Cofactor dependency	t_on_	Genetic payload	Biological origin	Host compatibility
AsLOV2	Conformational change	∼450 nm	Low	FMN (endogenous)	Fast	Small	Plant	Mammalian-compatible
LiD iLiD	Heterodimerization	∼450 nm	Low	FMN	Fast	Small	Bacterial	Mammalian-compatible
VVD	Homodimerization	∼450 nm	Low	FAD (endogenous)	Fast	Small	Fungal	Mammalian-compatible
NmPAL	Translation	∼450 nm	Low	FMN	Medium	Small	Bacterial	Limited in mammalian
EL222 YF1-FixJ	Transcription	∼450 nm	Low	FMN	Slow	Small/Large	Bacterial	Limited in mammalian
LOVTRAP cpLOV RsLOV	Dissociation	∼450 nm	Low	FMN	Fast	Small	Plant	Mammalian-compatible
CRY2-CRY2	Oligomerization	∼450 nm	Low	FAD (endogenous)	Fast	Moderate-Large	Plant	Mammalian-compatible
CRY2-CIB1	Heterodimerization	∼450 nm	Low	FAD	Fast	Moderate-Large	Plant	Mammalian-compatible
ChR2	Light-gated cation channel	∼472 nm	Low	Retinal (exogenous supplementation often required)	Fast	Small	Microbial	Mammalian-compatible
NpHR	Light-driven chloride pump	∼580 nm	Low	Retinal	Fast	Small	Microbial	Mammalian-compatible
Arch	Light-driven proton pump	∼550 nm	Low	Retinal	Medium	Small	Microbial	Mammalian-compatible
PhyB-PIF	Heterodimerization	∼650 nm	Moderate	PCB (exogenous)	Medium	Large	Plant	Limited in mammalian
BphP1/Q-PAS1	Heterodimerization	∼740 nm	High	Biliverdin (endogenous)	Medium	Large	Bacterial	Mammalian-compatible
BphS	c-di-GMP synthesis	∼730 nm	High	Biliverdin	Medium	Large	Bacterial	Mammalian-compatible

### 5.1 Clinical trial landscape for optogenetic cancer therapy

To date, no optogenetic cancer therapy has entered clinical trials, and most applications in oncology remain at the preclinical stage. In contrast, several opsin-based therapies have progressed to early-phase clinical trials for retinitis pigmentosa, demonstrating partial restoration of visual function (e.g., NCT02556736, NCT03326336, NCT05294978, and multiple other ongoing studies). This clinical progress is largely attributed to the availability of relatively safe gene delivery strategies and the anatomical accessibility of the retina to external light stimulation. By comparison, optogenetic cancer therapy faces more stringent challenges, including the need for efficient and targeted gene delivery as well as additional barriers related to light penetration in deep or heterogeneous tumor tissues.

Nevertheless, accumulating preclinical studies have provided strong proof-of-concept evidence, highlighting the potential of optogenetic approaches to advance toward early-phase clinical trials in oncology. Several key observations from the current clinical landscape are noteworthy: (1) AAV-mediated opsin gene delivery has been administered to patients with no severe adverse events, supporting the safety profile of viral-mediated photoreceptor delivery. (2) Clinical opsin studies rely on external illumination via goggles or implanted devices; analogous strategies for deep-seated tumors remain under development. (3) The established clinical infrastructure for CAR-T therapy provides a clear pathway for light-switchable CAR variants, with ongoing trials establishing dosing and monitoring protocols. (4) The FDA approval of NIR photoimmunotherapy (NIR-PIT, Akalux®) for head and neck cancer establishes a regulatory precedent for light-activated cancer therapies, which may inform the development of optogenetic products.

Despite these advances, the clinical translation of optogenetic cancer therapies still faces a long path ahead. Progress will require rigorous demonstration of safety and efficacy in large-animal models, the development of scalable manufacturing processes, and proactive engagement with regulatory agencies to establish appropriate clinical trial designs.

### 5.2 Technical limitations and optimization strategies

Despite the expanding repertoire of available OTs, many exhibit technical or structural limitations that constrain their utility *in vivo*. Context-specific optimization is therefore paramount to address these barriers and maximize therapeutic efficacy.

#### 5.2.1 Achieving unparalleled spatiotemporal precision

A cornerstone of optogenetics is its capacity for non-invasive and localized modulation of biological activity, which minimizes systemic toxicity. However, achieving this precision depends critically on the delivery modalities for both the optogenes and the activating light. Systemic administration of optogenetically engineered cells, such as CAR-T cells, often leads to widespread biodistribution, making it difficult to confine therapeutic effects to the tumor site. The precision of light delivery varies significantly by modality. While FUS or magnetic fields can penetrate deep tissues, their focal volume is often larger than that of localized NIR illumination. Similarly, systemic delivery of bioluminescent substrates can compromise spatial specificity, as light emission may occur in off-target tissues expressing the luciferase.

Therefore, ensuring therapeutic precision necessitates a dual-pronged approach: either (i) achieving tissue-specific targeting of the optogenetic constructs (e.g., using tumor-specific promoters or targeted viral vectors) or (ii) employing highly localized light delivery systems capable of precise volumetric illumination.

#### 5.2.2 Optimizing kinetic profiles and output efficiency

Therapeutic outcomes are closely tied to the activation/deactivation kinetics and dynamic range of OTs. The ideal tool is highly context-dependent. Systems with rapid on/off switching, such as those based on AsLOV2 or CRY2 oligomerization, are well-suited for applications requiring acute temporal control, such as immune cell activation or transient signaling modulation. In contrast, OTs with slower off-kinetics (e.g., VVD) support sustained outputs with minimal light exposure, suitable for prolonged gene expression or TME remodeling. A significant concern for many transcriptional systems is their requirement for prolonged illumination (hours), which raises issues of phototoxicity and thermal damage.

An optimal OT should exhibit low background activity (below the activation threshold) and achieves maximal output. For instance, the HSF system shows a higher baseline signal (∼10%) compared to LOV or CRY2 but maintains therapeutic efficacy due to high activation intensity (∼50%) [80]. Background activity and activation thresholds vary across cell types and signaling cascades, necessitating context-specific evaluation. In single-component systems, mutations that stabilize dark-state docking (e.g., I532A in LOV2) or modulate the interaction interface (e.g., electrostatic tuning in CRY2) can reduce background activity and enhance signal-to-noise ratio. For dual-component systems, interface-stabilizing or destabilizing mutations help improve efficiency.

#### 5.2.3 Bridging the cross-kingdom adaptation gap

Prokaryotic-derived OTs require extensive adaptation for mammalian application, addressing key factors such as cofactor availability, protein folding, post-translational modifications (e.g., phosphorylation, glycosylation), and subcellular localization. Each of these parameters critically influences functional integrity in eukaryotic contexts. For instance, cytoplasmic transcription systems such as EL222 and YF1–FixJ often require multiple mutations to enable efficient nuclear translocation without compromising light responsiveness—a process that remains technically challenging.

A powerful and increasingly popular strategy to circumvent these limitations is to bypass cross-kingdom adaptation altogether by integrating prokaryotic OTs with bacterial therapy. This approach enables efficient *in vivo* deployment while preserving the native regulatory mechanisms of the prokaryotic system, representing a paradigm shift in translational tool development [170,184].

### 5.3 Safety considerations and mitigation strategies

The clinical translation of optogenetic systems requires careful evaluation of potential safety risks associated with light activation, exogenous protein expression, and delivery platforms. Although optogenetics offers precise spatiotemporal control, which theoretically enables improved safety compared with conventional systemic therapies, several challenges must be addressed to ensure safe and effective *in vivo* implementation.

A major concern is phototoxicity induced by light exposure, particularly under high-intensity or short-wavelength illumination. BL, commonly used for activating LOV- and CRY2-based systems, can generate ROS and induce cellular damage. Prolonged illumination required for transcriptional systems (hours) further exacerbates this risk. To mitigate phototoxicity, several strategies have been developed, including shifting activation wavelengths toward the red or NIR spectrum, reducing light intensity and exposure duration, and employing light-converting materials (e.g., UCNPs or mechanoluminescent systems) to minimize photodamage while maintaining activation efficiency.

Another important consideration is the immunogenicity of optogenetic components, many of which are derived from non-mammalian sources such as plants (e.g., LOV and CRY2 domains), bacteria (e.g., microbial opsins), or fungi (e.g., VVD). The expression of these exogenous photoreceptors may elicit host immune responses, particularly under conditions of long-term expression or repeated administration, potentially leading to reduced therapeutic efficacy or adverse immune reactions. Moreover, differences in protein structure and post-translational processing between non-mammalian and mammalian systems may further increase the risk of immune recognition. Potential strategies to mitigate immunogenicity include protein engineering to remove or mask immunogenic epitopes, humanization of optogenetic domains, and the development of minimally immunogenic variants. Additionally, restricting expression to target tissues through the use of cell-specific promoters, transient expression systems, or localized delivery approaches may help reduce systemic immune activation.

Unintended light exposure or nonspecific gene delivery may lead to activation of optogenetic systems in non-target tissues, posing an additional safety challenge. This risk can be mitigated through the integration of tumor-targeting strategies, including the use of tumor-specific promoters, ligand-mediated delivery systems, and spatially confined light activation using implantable or localized light sources.

The safety of delivery platforms must also be carefully considered. Viral vectors, while highly efficient, raise concerns regarding insertional mutagenesis and long-term expression, whereas nanoparticle-based systems may introduce issues related to biodistribution, clearance, and potential toxicity. The development of biodegradable materials, transient expression systems, and dose optimization strategies will be essential to balance therapeutic efficacy with safety.

Collectively, these considerations highlight that the safety profile of optogenetic cancer therapy is not determined solely by the optogenetic tools themselves, but rather by the integration of molecular design, delivery strategies, and light-control modalities. Continued advances in protein engineering and biomaterials are expected to further enhance the safety and clinical feasibility of optogenetic approaches in oncology.

### 5.4 Challenges and adaptations for systemic malignancies

The high spatial resolution makes optogenetics highly suitable for solid tumor treatment, where confined anatomical localization enables targeted illumination. In contrast, hematological malignancies such as leukemia and lymphoma present distinct challenges for optogenetic intervention due to their systemic distribution and lack of spatial confinement. Circulating tumor cells and disseminated disease sites preclude simple spatial targeting strategies that rely on localized illumination. Under such conditions, optogenetic strategies may shift from spatially restricted activation toward temporally controlled regulation to improve therapeutic safety. Several adaptation strategies have emerged to address this paradigm. For example, Zhou et al. developed an UCNP-assisted optogenetic system for lymphoma treatment, in which implantable UCNP-based hydrogels enable localized NIR activation. Circulating optogenetically engineered CAR-T cells are transiently activated only when passing through the illuminated region, thereby reducing off-target effects associated with continuous CAR-T activation [18]. For hematological malignancies amenable to stem cell transplantation, optogenetic modification and activation can be performed *ex vivo* prior to infusion, eliminating the need for *in vivo* light delivery [18,179]. This approach capitalizes on the temporal precision of optogenetic control while circumventing the spatial constraints imposed by systemic disease distribution.

## 6 Future perspective

To realize the full clinical potential of optogenetic cancer therapy, future efforts must coalesce around three synergistic pillars: (i) the development of integrated hybrid systems, (ii) the intelligent engineering of superior OTs, and (iii) the strategic repurposing and expansion of opsin-based OTs.

### 6.1 Hybrid systems

The development of hybrid systems that integrate optogenetics with complementary therapeutic modalities represents an important frontier in biomedical engineering. Biomaterial-assisted delivery has enabled the construction of multifunctional platforms, including optogenetic–photothermal, –photodynamic, and –bioimaging systems, which combine the spatial precision of optical control with the functional versatility of advanced materials. For example, NIR-activated photothermal agents can simultaneously induce HSF-mediated gene expression and achieve localized thermal ablation, thereby enhancing therapeutic efficacy. Beyond synthetic biomaterials, living systems provide an additional avenue for integration. Bacterial optogenetic platforms expand the available OT repertoire, and although some systems require exogenous cofactors (e.g., PadC–MrkH) [184], pre-loading these components during culture offers a feasible strategy for *in vivo* application. Furthermore, integration with established treatment modalities, such as chemotherapy, holds substantial translational potential by enabling spatiotemporally controlled activation of therapeutic responses while minimizing systemic toxicity.

### 6.2 Intelligent engineering of OTs

Artificial intelligence (AI) is transforming the engineering paradigm of OTs, driving a shift from empirical, trial-and-error optimization toward rational and data-driven design. Traditional approaches rely heavily on iterative mutagenesis and high-throughput screening, which are labor-intensive, time-consuming, and often yield unpredictable outcomes due to the complex coupling between protein structure, photophysics, and functional output. In contrast, AI-based strategies integrate large-scale structural, sequence, and functional datasets to identify critical residues, predict stabilizing or function-enhancing mutations, and model light-induced conformational dynamics with high resolution. Recent advances in protein structure prediction and generative modeling further enable the *de novo* design of optogenetic domains with tailored spectral properties, switching kinetics, and interaction interfaces. Moreover, machine learning-assisted analysis of key performance metrics such as dynamic range, background activity, and response kinetics, facilitates multi-parameter optimization that is challenging to achieve through conventional methods. By substantially narrowing the experimental search space and prioritizing high-probability variants, these computational approaches not only accelerate the development cycle of next-generation OTs but also open new opportunities for designing application-specific systems optimized for complex *in vivo* environments.

### 6.3 Neuromodulation and neuro–tumor interactions

A pragmatic and powerful translational strategy lies in repurposing optogenetic systems with established clinical credentials. Opsin-based tools, originally developed for neuromodulation, have achieved clinical success in neurological disorders, benefiting from well-characterized safety profiles and mature implementation technologies. These attributes make them attractive candidates for repurposing in cancer therapy, either through direct effects on tumor cells or indirect modulation of the tumor microenvironment.

Opsin-based systems can directly modulate intracellular physicochemical states in non-neuronal cells. For example, ChR2, widely used to regulate neuronal excitability via cation influx, can be leveraged in tumor cells to elevate intracellular Ca^2+^ levels, thereby activating oncological relevant pathways such as MAPK/ERK and Notch signaling. The ChR2 C128S/D156A mutant exhibits prolonged open-state kinetics, maintaining channel activity for minutes after illumination and thus reducing the requirement for continuous light exposure. Similarly, proton-pumping opsins such as ChR1 enable direct modulation of intracellular and extracellular pH. By disrupting the acidic adaptation characteristic of the TME, ChR1-based approaches may reduce drug efflux and therapeutic resistance [185]. When combined with conventional treatments, such pH modulation can sensitize tumor cells and enhance overall therapeutic efficacy. In recent years, the involvement of the nervous system in tumor progression has gained increasing recognition. Emerging evidence indicates that neural activity actively participates in tumor development, including tumor innervation and neural modulation of the TME. In this context, optogenetic control of neuronal circuits may influence tumor growth in a non-cell-autonomous manner. For instance, in certain cancers such as small cell lung cancer, neural infiltration into tumor tissues has been associated with tumor progression, suggesting that modulation of neural activity may serve as a complementary tumor-suppressive strategy. In glioblastoma, neuronal activity has been shown to directly affect glioma cell behavior through paracrine signaling and synaptic-like interactions, supporting the potential of optogenetic neuromodulation in tumor-associated neural circuits. By targeting specific neuronal populations or pathways, opsin-based tools offer the ability to disrupt pro-tumor neural signaling or enhance anti-tumor immune responses. Together, these direct and indirect mechanisms position opsin-based systems as versatile adjuncts to existing cancer therapies, expanding the translational potential of optogenetics beyond the direct manipulation of malignant cells. Continued exploration of neuro–tumor interactions is expected to uncover new therapeutic opportunities that leverage the clinical maturity of opsin technologies.

## 7 Conclusion

Optogenetics stands at the forefront of precision oncology, offering an unprecedented level of spatiotemporal control over therapeutic interventions. Although significant challenges remain in delivery, activation depth, kinetic optimization, safety, and adaptation to diverse tumor types, the path forward is being paved by rapid advances in protein engineering, nanotechnology, and hybrid therapeutic platforms. The clinical precedent established by opsin-based therapies in neurology and ophthalmology, combined with the growing preclinical validation of non-opsin systems in oncology, provides a strong foundation for translation. Through continued integration of these interdisciplinary innovations, the field is moving toward the development of safe, effective, and minimally invasive optogenetic treatments with the potential to transform cancer care.

## CRediT authorship contribution statement

**Bing Yang:** Writing – original draft, Investigation, Conceptualization. **Qiyi Feng:** Supervision. **Chunxiu Xiao:** Supervision. **Xiuli Zheng:** Supervision. **Jingyao Chen:** Supervision. **Shuwen Xiao:** Supervision. **Zhichang Liu:** Supervision. **Kai Xiao:** Writing – review & editing, Supervision.

## Ethics approval and consent to participate

Not applicable. This article does not involve any studies with human participants or animals performed by any of the authors.

## Declaration of competing interest

The authors declare that they have no known competing financial interests or personal relationships that could have appeared to influence the work reported in this paper.

## Figures and Tables

**Table:** 

AAV	adeno-associated virus
**AH**	amphipathic helix
**AI**	artificial intelligence
**bHLH**	basic helix-loop-helix
**BL**	blue light
**BphPs**	bacterial phytochromes
**CAR**	chimeric antigen receptor
**CCT**	C-terminal extension
**cpLID**	circular permuted LID
**cpLOV2**	circularly permuted LOV2
**CRY2**	Cryptochrome 2
**EM**	effector module
**EPR**	enhanced permeability and retention
**ESA**	excited-state absorption
**FA**	folate acid
**FAD**	flavin adenine dinucleotide
**FKF1**	Flavin-Binding Kelch Repeat F-Box 1
**FLICs**	far-red light-controlled immunomodulatory engineered cells
**FMN**	flavin mononucleotide
**FOD**	flexible optoelectronic device
**FUS**	focused ultrasound
**Fz**	furimazine
**FFz**	fluorofurimazine
**GI**	GIGANTEA
**H-H-H**	Helix-Hairpin-Helix
**HK**	histidine kinase
**HRP**	horseradish peroxidase
**HSEs**	heat shock elements
**HSF**	heat shock factor
**HSPs**	heat shock proteins
**HTH**	helix-turn-helix
**iLID**	improved light-induced dimer
***i.t.***	intratumoral
***i.v.***	intravenous
**LAB**	*lactic acid bacteria*
**LiCAR**	light-switchable CAR
**LiTE**	light-inducible T cell engager
**LNPs**	lipid nanoparticles
**Ln^3+^**	lanthanide ions
**LOV**	light-oxygen-voltage
**LOVTRAP**	LOV2 Trap and Release of Protein
**MF**	magnetic field
**Ncap**	N-terminal A’α helix
**NES**	nuclear export signal
**NIR**	near-infrared
**NK**	natural killing
**NLS**	nuclear localization signal
**OS**	optical stimulation
**OT**	optogenetic tool
**PAS**	Per-ARNT-Sim
**PEI**	polyethyleneimine
**PHR**	photolyase-homologous region
**PLGA**	poly (lactic-co-glycolic acid)
**PM**	plasma membrane
**POI**	protein of interest
**PSM**	photosensitive module
**RBS**	ribosome-binding site
**RF**	radiofrequency
**RNP**	ribonucleoprotein
**ROS**	reactive oxygen species
**RsLOV**	*Rhodobacter sphaeroides* LOV
**scFv**	single-chain variable fragment
**SD**	Shine-Dalgarno
**STING**	stimulator of interferon genes
**TCR**	T cell receptor
**TEM**	Transmission electron microscope
**TF**	transcriptional factors
**TME**	tumor microenvironments
**UCL**	upconversion luminescence
**UCNPs**	upconversion nanoparticles
**UV**	ultraviolet
**VVD**	*Neurospora crassa* Vivid
**WT**	wild-type

## References

[1] Singh M., Overwijk W.W. (2015). Intratumoral immunotherapy for melanoma. Cancer Immunol. Immunother..

[2] Hoos A. (2016). Development of immuno-oncology drugs - from CTLA4 to PD1 to the next generations. Nat. Rev. Drug Discov..

[3] Sharma P., Hu-Lieskovan S., Wargo J.A., Ribas A. (2017). Primary, adaptive, and acquired resistance to cancer immunotherapy. Cell.

[4] Deisseroth K. (2011). Optogenetics. Nat. Methods.

[5] Kim T.I., McCall J.G., Jung Y.H., Huang X., Siuda E.R., Li Y., Song J., Song Y.M., Pao H.A., Kim R.H., Lu C., Lee S.D., Song I.S., Shin G., Al-Hasani R., Kim S., Tan M.P., Huang Y., Omenetto F.G., Rogers J.A., Bruchas M.R. (2013). Injectable, cellular-scale optoelectronics with applications for wireless optogenetics. Science.

[6] Pathak G.P., Spiltoir J.I., Höglund C., Polstein L.R., Heine-Koskinen S., Gersbach C.A., Rossi J., Tucker C.L. (2017). Bidirectional approaches for optogenetic regulation of gene expression in mammalian cells using Arabidopsis cryptochrome 2. Nucleic Acids Res..

[7] Wykes R.C., Heeroma J.H., Mantoan L., Zheng K., MacDonald D.C., Deisseroth K., Hashemi K.S., Walker M.C., Schorge S., Kullmann D.M. (2012). Optogenetic and potassium channel gene therapy in a rodent model of focal neocortical epilepsy. Sci. Transl. Med..

[8] Chaudhury D., Walsh J.J., Friedman A.K., Juarez B., Ku S.M., Koo J.W., Ferguson D., Tsai H.C., Pomeranz L., Christoffel D.J., Nectow A.R., Ekstrand M., Domingos A., Mazei-Robison M.S., Mouzon E., Lobo M.K., Neve R.L., Friedman J.M., Russo S.J., Deisseroth K., Nestler E.J., Han M.H. (2013). Rapid regulation of depression-related behaviours by control of midbrain dopamine neurons. Nature.

[9] Abdo H., Calvo-Enrique L., Lopez J.M., Song J., Zhang M.D., Usoskin D., El Manira A., Adameyko I., Hjerling-Leffler J., Ernfors P. (2019). Specialized cutaneous schwann cells initiate pain sensation. Science.

[10] Luo L., Callaway E.M., Svoboda K. (2018). Genetic dissection of neural circuits: a decade of progress. Neuron.

[11] Miura Y., Li M.Y., Revah O., Yoon S.J., Narazaki G., Pasca S.P. (2022). Engineering brain assembloids to interrogate human neural circuits. Nat. Protoc..

[12] Saunders B.T., Richard J.M., Margolis E.B., Janak P.H. (2018). Dopamine neurons create pavlovian conditioned stimuli with circuit-defined motivational properties. Nat. Neurosci..

[13] Tonegawa S., Liu X., Ramirez S., Redondo R. (2015). Memory engram cells have come of age. Neuron.

[14] Lu Q., Sun Y., Liang Z., Zhang Y., Wang Z., Mei Q. (2024). Nano-optogenetics for disease therapies. ACS Nano.

[15] Malogolovkin A., Egorov A.D., Karabelsky A., Ivanov R.A., Verkhusha V.V. (2022). Optogenetic technologies in translational cancer research. Biotechnol. Adv..

[16] Manoilov K.Y., Verkhusha V.V., Shcherbakova D.M. (2021). A guide to the optogenetic regulation of endogenous molecules. Nat. Methods.

[17] Lorenzo-Martin L.F., Hubscher T., Bowler A.D., Broguiere N., Langer J., Tillard L., Nikolaev M., Radtke F., Lutolf M.P. (2024). Spatiotemporally resolved colorectal oncogenesis in mini-colons ex vivo. Nature.

[18] Nguyen N.T., Huang K., Zeng H., Jing J., Wang R., Fang S., Chen J., Liu X., Huang Z., You M.J., Rao A., Huang Y., Han G., Zhou Y. (2021). Nano-optogenetic engineering of CAR T cells for precision immunotherapy with enhanced safety. Nat. Nanotechnol..

[19] Gligorovski V., Sadeghi A., Rahi S.J. (2023). Multidimensional characterization of inducible promoters and a highly light-sensitive LOV-transcription factor. Nat. Commun..

[20] Glantz S.T., Carpenter E.J., Melkonian M., Gardner K.H., Boyden E.S., Wong G.K., Chow B.Y. (2016). Functional and topological diversity of LOV domain photoreceptors. Proc. Natl. Acad. Sci. U. S. A..

[21] Vlasova A.D., Bukhalovich S.M., Bagaeva D.F., Polyakova A.P., Ilyinsky N.S., Nesterov S.V., Tsybrov F.M., Bogorodskiy A.O., Zinovev E.V., Mikhailov A.E., Vlasov A.V., Kuklin A.I., Borshchevskiy V.I., Bamberg E., Uversky V.N., Gordeliy V.I. (2024). Intracellular microbial rhodopsin-based optogenetics to control metabolism and cell signaling. Chem. Soc. Rev..

[22] Cardin J.A., Carlén M., Meletis K., Knoblich U., Zhang F., Deisseroth K., Tsai L.H., Moore C.I. (2010). Targeted optogenetic stimulation and recording of neurons in vivo using cell-type-specific expression of Channelrhodopsin-2. Nat. Protoc..

[23] Emiliani V., Entcheva E., Hedrich R., Hegemann P., Konrad K.R., Luscher C., Mahn M., Pan Z.H., Sims R.R., Vierock J., Yizhar O. (2022). Optogenetics for light control of biological systems. Nat. Rev. Methods Primers.

[24] Tischer D., Weiner O.D. (2014). Illuminating cell signalling with optogenetic tools. Nat. Rev. Mol. Cell Biol..

[25] Huang K., Liu L., Huang Y., Wang Y., Zhou Y., Han G. (2023). Remote control of cellular immunotherapy. Nat. Rev. Bioeng..

[26] Tan P., He L., Han G., Zhou Y. (2017). Optogenetic immunomodulation: shedding light on antitumor immunity. Trends Biotechnol..

[27] Lan T.H., He L., Huang Y., Zhou Y. (2022). Optogenetics for transcriptional programming and genetic engineering. Trends Genet..

[28] Zhou X.X., Fan L.Z., Li P., Shen K., Lin M.Z. (2017). Optical control of cell signaling by single-chain photoswitchable kinases. Science.

[29] Khanna R., Huq E., Kikis E.A., Al-Sady B., Lanzatella C., Quail P.H. (2004). A novel molecular recognition motif necessary for targeting photoactivated phytochrome signaling to specific basic helix-loop-helix transcription factors. Plant Cell.

[30] Kennis J.T., Crosson S., Gauden M., van Stokkum I.H., Moffat K., van Grondelle R. (2003). Primary reactions of the LOV2 domain of phototropin, a plant blue-light photoreceptor. Biochemistry.

[31] Arshi S.A., Chauhan M., Sharma A. (2023). Disruption of the FMN-A524 interaction cascade and Glu513-induced collapse of the hydrophobic barrier promotes light-induced Jalpha-helix unfolding in AsLOV2. Biophys. J..

[32] Harper S.M., Neil L.C., Gardner K.H. (2003). Structural basis of a phototropin light switch. Science.

[33] Harper S.M., Neil L.C., Gardner K.H. (2003). Structural basis of a phototropin light switch. Science.

[34] Xiao S., Ibrahim M.T., Verkhivker G.M., Zoltowski B.D., Tao P. (2024). beta-sheets mediate the conformational change and allosteric signal transmission between the AsLOV2 termini. J. Comput. Chem..

[35] Strickland D., Yao X., Gawlak G., Rosen M.K., Gardner K.H., Sosnick T.R. (2010). Rationally improving LOV domain-based photoswitches. Nat. Methods.

[36] Stone O.J., Pankow N., Liu B., Sharma V.P., Eddy R.J., Wang H., Putz A.T., Teets F.D., Kuhlman B., Condeelis J.S., Hahn K.M. (2019). Optogenetic control of cofilin and alphaTAT in living cells using Z-lock. Nat. Chem. Biol..

[37] Wang H., Vilela M., Winkler A., Tarnawski M., Schlichting I., Yumerefendi H., Kuhlman B., Liu R., Danuser G., Hahn K.M. (2016). LOVTRAP: an optogenetic system for photoinduced protein dissociation. Nat. Methods.

[38] Lungu O.I., Hallett R.A., Choi E.J., Aiken M.J., Hahn K.M., Kuhlman B. (2012). Designing photoswitchable peptides using the AsLOV2 domain. Chem. Biol..

[39] Guntas G., Hallett R.A., Zimmerman S.P., Williams T., Yumerefendi H., Bear J.E., Kuhlman B. (2015). Engineering an improved light-induced dimer (iLID) for controlling the localization and activity of signaling proteins. Proc. Natl. Acad. Sci. U. S. A..

[40] He L., Tan P., Zhu L., Huang K., Nguyen N.T., Wang R., Guo L., Li L., Yang Y., Huang Z., Huang Y., Han G., Wang J., Zhou Y. (2021). Circularly permuted LOV2 as a modular photoswitch for optogenetic engineering. Nat. Chem. Biol..

[41] Yao Y., Lou X., Jin L., Sun W., Liu J., Chen Y., Cheng S., Zhao T., Ke S., Zhang L., Xu Y., He L., Li H. (2024). Optogenetic strategies for optimizing the performance of phospholipids biosensors. Adv. Sci. (Weinh.).

[42] Zoltowski B.D., Schwerdtfeger C., Widom J., Loros J.J., Bilwes A.M., Dunlap J.C., Crane B.R. (2007). Conformational switching in the fungal light sensor vivid. Science.

[43] Kawano F., Suzuki H., Furuya A., Sato M. (2015). Engineered pairs of distinct photoswitches for optogenetic control of cellular proteins. Nat. Commun..

[44] Zoltowski B.D., Vaccaro B., Crane B.R. (2009). Mechanism-based tuning of a LOV domain photoreceptor. Nat. Chem. Biol..

[45] Liu R., Yang J., Yao J., Zhao Z., He W., Su N., Zhang Z., Zhang C., Zhang Z., Cai H., Zhu L., Zhao Y., Quan S., Chen X., Yang Y. (2022). Optogenetic control of RNA function and metabolism using engineered light-switchable RNA-binding proteins. Nat. Biotechnol..

[46] Yazawa M., Sadaghiani A.M., Hsueh B., Dolmetsch R.E. (2009). Induction of protein-protein interactions in live cells using light. Nat. Biotechnol..

[47] Pal A.A., Benman W., Mumford T.R., Huang Z., Chow B.Y., Bugaj L.J. (2023). Optogenetic clustering and membrane translocation of the BcLOV4 photoreceptor. Proc. Natl. Acad. Sci. U. S. A..

[48] Ji Y., Heidari A., Nzigou Mombo B., Wegner S.V. (2024). Photoactivation of LOV domains with chemiluminescence. Chem. Sci..

[49] Glantz S.T., Berlew E.E., Jaber Z., Schuster B.S., Gardner K.H., Chow B.Y. (2018). Directly light-regulated binding of RGS-LOV photoreceptors to anionic membrane phospholipids. Proc. Natl. Acad. Sci. U. S. A..

[50] Berlew E.E., Kuznetsov I.A., Yamada K., Bugaj L.J., Boerckel J.D., Chow B.Y. (2021). Single-component optogenetic tools for inducible RhoA GTPase signaling. Adv. Biol. (Weinh).

[51] Benman W., Berlew E.E., Deng H., Parker C., Kuznetsov I.A., Lim B., Siekmann A.F., Chow B.Y., Bugaj L.J. (2022). Temperature-responsive optogenetic probes of cell signaling. Nat. Chem. Biol..

[52] Conrad K.S., Bilwes A.M., Crane B.R. (2013). Light-induced subunit dissociation by a light-oxygen-voltage domain photoreceptor from Rhodobacter sphaeroides. Biochemistry.

[53] Nash A.I., McNulty R., Shillito M.E., Swartz T.E., Bogomolni R.A., Luecke H., Gardner K.H. (2011). Structural basis of photosensitivity in a bacterial light-oxygen-voltage/helix-turn-helix (LOV-HTH) DNA-binding protein. Proc. Natl. Acad. Sci. U. S. A..

[54] Motta-Mena L.B., Reade A., Mallory M.J., Glantz S., Weiner O.D., Lynch K.W., Gardner K.H. (2014). An optogenetic gene expression system with rapid activation and deactivation kinetics. Nat. Chem. Biol..

[55] Takakado A., Nakasone Y., Terazima M. (2017). Photoinduced dimerization of a photosensory DNA-binding protein EL222 and its LOV domain. Phys. Chem. Chem. Phys..

[56] Giniger E., Varnum S.M., Ptashne M. (1985). Specific DNA binding of GAL4, a positive regulatory protein of yeast. Cell.

[57] Takakado A., Nakasone Y., Terazima M. (2018). Sequential DNA binding and dimerization processes of the photosensory protein EL222. Biochemistry.

[58] Yang C., Cui M., Zhang Y., Pan H., Liu J., Wang S., Ma N., Chang J., Sun T., Wang H. (2020). Upconversion optogenetic micro-nanosystem optically controls the secretion of light-responsive bacteria for systemic immunity regulation. Commun. Biol..

[59] Ohlendorf R., Vidavski R.R., Eldar A., Moffat K., Möglich A. (2012). From dusk till dawn: one-plasmid systems for light-regulated gene expression. J. Mol. Biol..

[60] Möglich A., Ayers R.A., Moffat K. (2009). Design and signaling mechanism of light-regulated histidine kinases. J. Mol. Biol..

[61] Weber A.M., Kaiser J., Ziegler T., Pilsl S., Renzl C., Sixt L., Pietruschka G., Moniot S., Kakoti A., Juraschitz M., Schrottke S., Lledo Bryant L., Steegborn C., Bittl R., Mayer G., Möglich A. (2019). A blue light receptor that mediates RNA binding and translational regulation. Nat. Chem. Biol..

[62] Ranzani A.T., Buchholz K., Blackholm M., Kopkin H., Möglich A. (2024). Induction of bacterial expression at the mRNA level by light. Nucleic Acids Res..

[63] Ahmad M., Cashmore A.R. (1993). HY4 gene of A. thaliana encodes a protein with characteristics of a blue-light photoreceptor. Nature.

[64] Ma L., Guan Z., Wang Q., Yan X., Wang J., Wang Z., Cao J., Zhang D., Gong X., Yin P. (2020). Structural insights into the photoactivation of Arabidopsis CRY2. Nat. Plants.

[65] Park H., Kim N.Y., Lee S., Kim N., Kim J., Heo W.D. (2017). Optogenetic protein clustering through fluorescent protein tagging and extension of CRY2. Nat. Commun..

[66] Palayam M., Ganapathy J., Guercio A.M., Tal L., Deck S.L., Shabek N. (2021). Structural insights into photoactivation of plant Cryptochrome-2. Commun. Biol..

[67] Lee S., Park H., Kyung T., Kim N.Y., Kim S., Kim J., Heo W.D. (2014). Reversible protein inactivation by optogenetic trapping in cells. Nat. Methods.

[68] Taslimi A., Vrana J.D., Chen D., Borinskaya S., Mayer B.J., Kennedy M.J., Tucker C.L. (2014). An optimized optogenetic clustering tool for probing protein interaction and function. Nat. Commun..

[69] He L., Huang Z., Huang K., Chen R., Nguyen N.T., Wang R., Cai X., Huang Z., Siwko S., Walker J.R., Han G., Zhou Y., Jing J. (2021). Optogenetic control of non-apoptotic cell death. Adv. Sci. (Weinh.).

[70] Taslimi A., Fields K.M., Dahl K.D., Liu Q., Tucker C.L. (2022). Spatiotemporal control of necroptotic cell death and plasma membrane recruitment using engineered MLKL domains. Cell Death Discov..

[71] Park H., Kim N.Y., Lee S., Kim N., Kim J., Heo W.D. (2017). Optogenetic protein clustering through fluorescent protein tagging and extension of CRY2. Nat. Commun..

[72] Kim S., Kyung T., Chung J.H., Kim N., Keum S., Lee J., Park H., Kim H.M., Lee S., Shin H.S., Heo W.D. (2020). Non-invasive optical control of endogenous Ca(2+) channels in awake mice. Nat. Commun..

[73] Oh T.J., Krishnamurthy V., Han J.W., Zhu J., Beg Z., Mehfooz A., Gworek B., Shapiro D.J., Zhang K. (2024). Spatiotemporal control of inflammatory lytic cell death through optogenetic induction of RIPK3 oligomerization. J. Mol. Biol..

[74] Huang Z., Wu Y., Allen M.E., Pan Y., Kyriakakis P., Lu S., Chang Y.J., Wang X., Chien S., Wang Y. (2020). Engineering light-controllable CAR T cells for cancer immunotherapy. Sci. Adv..

[75] Hao Y., Zhang X., Liu Y., Ma M., Huang X., Liu H., Zhang P. (2023). Cryo-EM structure of the CRY2 and CIB1 fragment complex provides insights into CIB1-mediated photosignaling. Plant Commun..

[76] Liu H., Yu X., Li K., Klejnot J., Yang H., Lisiero D., Lin C. (2008). Photoexcited CRY2 interacts with CIB1 to regulate transcription and floral initiation in Arabidopsis. Science.

[77] Kennedy M.J., Hughes R.M., Peteya L.A., Schwartz J.W., Ehlers M.D., Tucker C.L. (2010). Rapid blue-light-mediated induction of protein interactions in living cells. Nat. Methods.

[78] Fu X., Huang Y., Zhao H., Zhang E., Shen Q., Di Y., Lv F., Liu L., Wang S. (2021). Near-infrared-light remote-controlled activation of cancer immunotherapy using photothermal conjugated polymer nanoparticles. Adv. Mater..

[79] Wang Y., Li S., Zhang P., Bai H., Feng L., Lv F., Liu L., Wang S. (2018). Photothermal-responsive conjugated polymer nanoparticles for remote control of gene expression in living cells. Adv. Mater..

[80] Chen X., Chen Y., Xin H., Wan T., Ping Y. (2020). Near-infrared optogenetic engineering of photothermal nanoCRISPR for programmable genome editing. Proc. Natl. Acad. Sci. U. S. A..

[81] Lyu Y., Cui D., Sun H., Miao Y., Duan H., Pu K. (2017). Dendronized semiconducting polymer as photothermal nanocarrier for remote activation of gene expression. Angew Chem. Int. Ed. Engl..

[82] Miller I.C., Zamat A., Sun L.K., Phuengkham H., Harris A.M., Gamboa L., Yang J., Murad J.P., Priceman S.J., Kwong G.A. (2021). Enhanced intratumoural activity of CAR T cells engineered to produce immunomodulators under photothermal control. Nat. Biomed. Eng..

[83] Wu Y., Liu Y., Huang Z., Wang X., Jin Z., Li J., Limsakul P., Zhu L., Allen M., Pan Y., Bussell R., Jacobson A., Liu T., Chien S., Wang Y. (2021). Control of the activity of CAR-T cells within tumours via focused ultrasound. Nat. Biomed. Eng..

[84] Toh P.J.Y., Lai J.K.H., Hermann A., Destaing O., Sheetz M.P., Sudol M., Saunders T.E. (2022). Optogenetic control of YAP cellular localisation and function. EMBO Rep..

[85] Nzigou Mombo B., Bijonowski B.M., Raab C.A., Niland S., Brockhaus K., Muller M., Eble J.A., Wegner S.V. (2023). Reversible photoregulation of cell-cell adhesions with opto-E-cadherin. Nat. Commun..

[86] Nagashima Y., Eguchi T., Koyama-Honda I., Mizushima N. (2025). Optogenetic tools for inducing organelle membrane rupture. J. Biol. Chem..

[87] He L., Wang L., Zeng H., Tan P., Ma G., Zheng S., Li Y., Sun L., Dou F., Siwko S., Huang Y., Wang Y., Zhou Y. (2021). Engineering of a bona fide light-operated calcium channel. Nat. Commun..

[88] Ueda Y., Miura Y., Tomishige N., Sugimoto N., Murase M., Kawamura G., Sasaki N., Ishiwata T., Ozawa T. (2022). Mechanistic insights into cancer drug resistance through optogenetic PI3K signaling hyperactivation. Cell Chem. Biol..

[89] de Seze J., Bongaerts M., Boulevard B., Coppey M. (2025). Optogenetic control of a GEF of RhoA uncovers a signaling switch from retraction to protrusion. eLife.

[90] Lee Y.T., Guo L., Lan T.H., Nonomura T., Liu G., Ma G., Wang R., Huang Y., Zhou Y. (2025). Engineering of photo-inducible binary interaction tools for biomedical applications. Nat. Commun..

[91] Kalafut J., Czapinski J., Przybyszewska-Podstawka A., Czerwonka A., Odrzywolski A., Sahlgren C., Rivero-Muller A. (2022). Optogenetic control of NOTCH1 signaling. Cell Commun. Signal..

[92] Pan H., Wang H., Yu J., Huang X., Hao Y., Zhang C., Ji W., Yang M., Gong X., Wu X., Chang J. (2019). Near-infrared light remotely up-regulate autophagy with spatiotemporal precision via upconversion optogenetic nanosystem. Biomaterials.

[93] Zhou X., Wang J., Chen J., Qi Y., Di N., Jin L., Qian X., Wang X., Chen Q., Liu X., Xu Y. (2018). Optogenetic control of epithelial-mesenchymal transition in cancer cells. Sci. Rep..

[94] Lin F., Dong L., Wang W., Liu Y., Huang W., Cai Z. (2016). An efficient light-inducible P53 expression system for inhibiting proliferation of bladder cancer cell. Int. J. Biol. Sci..

[95] Wend S., Wagner H.J., Muller K., Zurbriggen M.D., Weber W., Radziwill G. (2014). Optogenetic control of protein kinase activity in mammalian cells. ACS Synth. Biol..

[96] Mo W., Su S., Shang R., Yang L., Zhao X., Wu C., Yang Z., Zhang H., Wu L., Liu Y., He Y., Zhang R., Zuo Z. (2023). Optogenetic induction of caspase-8 mediated apoptosis by employing Arabidopsis cryptochrome 2. Sci. Rep..

[97] Tan P., He L., Zhou Y. (2021). Engineering supramolecular organizing centers for optogenetic control of innate immune responses. Adv. Biol. (Weinh).

[98] Jeong D.H., Kim S., Park H.H., Woo K.J., Choi J.I., Choi M., Shin J., Park S.H., Seon M.W., Lee D., Cha J.H., Kim Y.S. (2025). Optogenetically activatable MLKL as a standalone functional module for necroptosis and therapeutic applications in antitumoral immunity. Adv. Sci. (Weinh.).

[99] Bugaj L.J., Sabnis A.J., Mitchell A., Garbarino J.E., Toettcher J.E., Bivona T.G., Lim W.A. (2018). Cancer mutations and targeted drugs can disrupt dynamic signal encoding by the Ras-Erk pathway. Science.

[100] Gil A.A., Laptenok S.P., French J.B., Iuliano J.N., Lukacs A., Hall C.R., Sazanovich I.V., Greetham G.M., Bacher A., Illarionov B., Fischer M., Tonge P.J., Meech S.R. (2017). Femtosecond to millisecond dynamics of light induced allostery in the Avena sativa LOV domain. J. Phys. Chem. B.

[101] Raffelberg S., Mansurova M., Gartner W., Losi A. (2011). Modulation of the photocycle of a LOV domain photoreceptor by the hydrogen-bonding network. J. Am. Chem. Soc..

[102] Duan L., Hope J., Ong Q., Lou H.Y., Kim N., McCarthy C., Acero V., Lin M.Z., Cui B. (2017). Understanding CRY2 interactions for optical control of intracellular signaling. Nat. Commun..

[103] !!! INVALID CITATION !!!).

[104] Wang X., Chen X., Yang Y. (2012). Spatiotemporal control of gene expression by a light-switchable transgene system. Nat. Methods.

[105] Fu X.C., Huang Y.M., Zhao H., Zhang E.D., Shen Q., Di Y.F., Lv F.T., Liu L.B., Wang S. (2021). Near-infrared-light remote-controlled activation of cancer immunotherapy using photothermal conjugated polymer nanoparticles. Adv. Mater..

[106] Yao X., Rosen M.K., Gardner K.H. (2008). Estimation of the available free energy in a LOV2-J alpha photoswitch. Nat. Chem. Biol..

[107] Zayner J.P., Mathes T., Sosnick T.R., Kennis J.T.M. (2019). Helical contributions mediate light-activated conformational change in the LOV2 domain of Avena sativa phototropin 1. ACS Omega.

[108] Kyung T., Lee S., Kim J.E., Cho T., Park H., Jeong Y.M., Kim D., Shin A., Kim S., Baek J., Kim J., Kim N.Y., Woo D., Chae S., Kim C.H., Shin H.S., Han Y.M., Kim D., Heo W.D. (2015). Optogenetic control of endogenous Ca(2+) channels in vivo. Nat. Biotechnol..

[109] Hagihara Y., Sakamoto A., Tokuda T., Yamashita T., Ikemoto S., Kimura A., Haruta M., Sasagawa K., Ohta J., Takayama K., Mizuguchi H. (2020). Photoactivatable oncolytic adenovirus for optogenetic cancer therapy. Cell Death Dis..

[110] Kamiya H., Tsuchiya H., Yamazaki J., Harashima H. (2001). Intracellular trafficking and transgene expression of viral and non-viral gene vectors. Adv. Drug Deliv. Rev..

[111] Lee Y., Kim B., Lee D., Cheon S.Y., Lim S.G., Kim Y., Koo H. (2025). In vivo delivery systems for CRISPR genome editing: viral and non-viral carriers. Appl. Phys. Rev..

[112] Gong F., Li B. (2024). Hybrid non-viral and viral delivery strategy achieves potent gene editing in growing livers with reduced viral dosage. Mol. Ther..

[113] Sun X., Tian T., Lian Y., Cui Z. (2024). Current advances in viral nanoparticles for biomedicine. ACS Nano.

[114] Sato M., Inada E., Saitoh I., Watanabe S., Nakamura S. (2020). piggyBac-Based non-viral in vivo gene delivery useful for production of genetically modified animals and organs. Pharmaceutics.

[115] Burns K.H., Boeke J.D. (2012). Human transposon tectonics. Cell.

[116] Singh M., Mazaheri-Tehrani G., Martin-Fabiani I., Davies O.G. (2025). Electroporation induced changes in extracellular vesicle profile. Drug Deliv..

[117] Manion M.L., Liu A.T. (2024). DNA augmented intracellular protein delivery via nanopore electroporation. Matter.

[118] Wang J.H., Gessler D.J., Zhan W., Gallagher T.L., Gao G. (2024). Adeno-associated virus as a delivery vector for gene therapy of human diseases. Signal Transduct. Targeted Ther..

[119] Wu M., Li H., Zhang C., Wang Y., Zhang C., Zhang Y., Zhong A., Zhang D., Liu X. (2023). Silk-gel powered adenoviral vector enables robust genome editing of PD-L1 to augment immunotherapy across multiple tumor models. Adv. Sci. (Weinh.).

[120] Peng J., Liu K., Cao L., Duan D., Song G., Liu S., Wang L., Li J., Zhang X., Huang K., Zhao Y., Niu Y., Han G. (2022). Adenoviral vector for enhanced prostate cancer specific transferrin conjugated drug targeted therapy. Nano Lett..

[121] Wang C., Pan C., Yong H., Wang F., Bo T., Zhao Y., Ma B., He W., Li M. (2023). Emerging non-viral vectors for gene delivery. J. Nanobiotechnol..

[122] Mehta M., Bui T.A., Yang X., Aksoy Y., Goldys E.M., Deng W. (2023). Lipid-based nanoparticles for drug/gene delivery: an overview of the production techniques and difficulties encountered in their industrial development. ACS Mater. Au.

[123] Raguram A., Banskota S., Liu D.R. (2022). Therapeutic in vivo delivery of gene editing agents. Cell.

[124] Li J., Wang J., Chen Z. (2025). Emerging role of exosomes in cancer therapy: progress and challenges. Mol. Cancer.

[125] Cheng Z., Huang H., Yin M., Liu H. (2025). Applications of liposomes and lipid nanoparticles in cancer therapy: current advances and prospects. Exp. Hematol. Oncol..

[126] Hsieh F.Y., Han H.W., Chen X.R., Yang C.S., Wei Y., Hsu S.H. (2018). Non-viral delivery of an optogenetic tool into cells with self-healing hydrogel. Biomaterials.

[127] Kesharwani P., Iyer A.K. (2015). Recent advances in dendrimer-based nanovectors for tumor-targeted drug and gene delivery. Drug Discov. Today.

[128] Zhang Z., Wan T., Chen Y., Chen Y., Sun H., Cao T., Songyang Z., Tang G., Wu C., Ping Y., Xu F.J., Huang J. (2019). Cationic polymer-mediated CRISPR/Cas9 plasmid delivery for genome editing. Macromol. Rapid Commun..

[129] Ping Y., Hu Q., Tang G., Li J. (2013). FGFR-targeted gene delivery mediated by supramolecular assembly between beta-cyclodextrin-crosslinked PEI and redox-sensitive PEG. Biomaterials.

[130] Wang X., Zeng J., Gan D., Ling K., He M., Li J., Lu Y. (2024). Recent strategies and advances in hydrogel-based delivery platforms for bone regeneration. Nano-Micro Lett..

[131] Park S.I., Brenner D.S., Shin G., Morgan C.D., Copits B.A., Chung H.U., Pullen M.Y., Noh K.N., Davidson S., Oh S.J., Yoon J., Jang K.I., Samineni V.K., Norman M., Grajales-Reyes J.G., Vogt S.K., Sundaram S.S., Wilson K.M., Ha J.S., Xu R., Pan T., Kim T.I., Huang Y., Montana M.C., Golden J.P., Bruchas M.R., Gereau R.W.t., Rogers J.A. (2015). Soft, stretchable, fully implantable miniaturized optoelectronic systems for wireless optogenetics. Nat. Biotechnol..

[132] Montgomery K.L., Yeh A.J., Ho J.S., Tsao V., Mohan Iyer S., Grosenick L., Ferenczi E.A., Tanabe Y., Deisseroth K., Delp S.L., Poon A.S. (2015). Wirelessly powered, fully internal optogenetics for brain, spinal and peripheral circuits in mice. Nat. Methods.

[133] Wu M., Yang Y., Zhang J., Efimov A.I., Li X., Zhang K., Wang Y., Bodkin K.L., Riahi M., Gu J., Wang G., Kim M., Zeng L., Liu J., Yoon L.H., Zhang H., Freda S.N., Lee M., Kang J., Ciatti J.L., Ting K., Cheng S., Zhang X., Sun H., Zhang W., Zhang Y., Banks A., Good C.H., Cox J.M., Pinto L., Vazquez-Guardado A., Huang Y., Kozorovitskiy Y., Rogers J.A. (2026). Patterned wireless transcranial optogenetics generates artificial perception. Nat. Neurosci..

[134] Molkenova A., Choi H.E., Lee G., Baek H., Kwon M., Lee S.B., Park J.M., Kim J.H., Han D.W., Park J., Hahn S.K., Kim K.S. (2024). Cold-responsive hyaluronated upconversion nanoplatform for transdermal cryo-photodynamic cancer therapy. Adv. Sci. (Weinh.).

[135] Li D., Yu S.H., Jiang H.L. (2018). From UV to near-infrared light-responsive metal-organic framework composites: Plasmon and upconversion enhanced photocatalysis. Adv. Mater..

[136] Zhang H., Li Y., Ivanov I.A., Qu Y., Huang Y., Duan X. (2010). Plasmonic modulation of the upconversion fluorescence in NaYF4 :Yb/Tm hexaplate nanocrystals using gold nanoparticles or nanoshells. Angew Chem. Int. Ed. Engl..

[137] Chen S., Weitemier A.Z., Zeng X., He L., Wang X., Tao Y., Huang A.J.Y., Hashimotodani Y., Kano M., Iwasaki H., Parajuli L.K., Okabe S., Teh D.B.L., All A.H., Tsutsui-Kimura I., Tanaka K.F., Liu X., McHugh T.J. (2018). Near-infrared deep brain stimulation via upconversion nanoparticle-mediated optogenetics. Science.

[138] Wang Y., Lin X., Chen X., Chen X., Xu Z., Zhang W., Liao Q., Duan X., Wang X., Liu M., Wang F., He J., Shi P. (2017). Tetherless near-infrared control of brain activity in behaving animals using fully implantable upconversion microdevices. Biomaterials.

[139] Jeong J.W., McCall J.G., Shin G., Zhang Y., Al-Hasani R., Kim M., Li S., Sim J.Y., Jang K.I., Shi Y., Hong D.Y., Liu Y., Schmitz G.P., Xia L., He Z., Gamble P., Ray W.Z., Huang Y., Bruchas M.R., Rogers J.A. (2015). Wireless optofluidic systems for programmable in vivo pharmacology and optogenetics. Cell.

[140] Shin G., Gomez A.M., Al-Hasani R., Jeong Y.R., Kim J., Xie Z., Banks A., Lee S.M., Han S.Y., Yoo C.J., Lee J.L., Lee S.H., Kurniawan J., Tureb J., Guo Z., Yoon J., Park S.I., Bang S.Y., Nam Y., Walicki M.C., Samineni V.K., Mickle A.D., Lee K., Heo S.Y., McCall J.G., Pan T., Wang L., Feng X., Kim T.I., Kim J.K., Li Y., Huang Y., Gereau R.W.t., Ha J.S., Bruchas M.R., Rogers J.A. (2017). Flexible near-field wireless optoelectronics as subdermal implants for broad applications in optogenetics. Neuron.

[141] Geng S., Li H., Lv Z., Zhai Y., Tian B., Luo Y., Zhou Y., Han S.T. (2025). Challenges and opportunities of upconversion nanoparticles for emerging NIR optoelectronic devices. Adv. Mater..

[142] Schroter A., Arnau Del Valle C., Marín M.J., Hirsch T. (2023). Bilayer-coating strategy for hydrophobic nanoparticles providing colloidal stability, functionality, and surface protection in biological media. Angew Chem. Int. Ed. Engl..

[143] Wang F., Han Y., Lim C.S., Lu Y., Wang J., Xu J., Chen H., Zhang C., Hong M., Liu X. (2010). Simultaneous phase and size control of upconversion nanocrystals through lanthanide doping. Nature.

[144] Lin S., Liu W., Fu X., Luo M., Liu H., Zhong W.-H. (2023). An all-protein aerogel with a nanofiber/foam structure for versatile air filtration. Mater. Today Chem..

[145] Yang T., Wu Q., Dai F., Huang K., Xu H., Liu C., Chen C., Hu S., Liang X., Liu X., Noh Y.Y., Liu C. (2019). Understanding, optimizing, and utilizing nonideal transistors based on organic or organic hybrid semiconductors. Adv. Funct. Mater..

[146] Wang Y., Wang Y., Zhong H., Xiong L., Song J., Zhang X., He T., Zhou X., Li L., Zhen D. (2024). Recent progress of UCNPs-MoS(2) nanocomposites as a platform for biological applications. J. Mater. Chem. B.

[147] He X., Luo Y., Li Y., Pan Y., Kwok R.T.K., He L., Duan X., Zhang P., Wu A., Tang B.Z., Li J. (2023). D‐type neuropeptide decorated AIEgen/RENP hybrid nanoprobes with light‐driven ROS generation ability for NIR‐II fluorescence imaging‐guided through‐skull photodynamic therapy of gliomas. Aggregate.

[148] Guo Y., Zou R., Si F., Liang W., Zhang T., Chang Y., Qiao X., Zhao J. (2021). A sensitive immunoassay based on fluorescence resonance energy transfer from up-converting nanoparticles and graphene oxide for one-step detection of imidacloprid. Food Chem..

[149] Li J., Hou W., Lin S., Wang L., Pan C., Wu F., Liu J. (2022). Polydopamine nanoparticle-mediated dopaminergic immunoregulation in colitis. Adv. Sci. (Weinh.).

[150] Li Y., Yang B., Wang Y., Huang Z., Wang J., Pu X., Wen J., Ao Q., Xiao K., Wu J., Yin G. (2024). Postoperatively noninvasive optogenetic stimulation via upconversion nanoparticles enhancing sciatic nerve repair. Nano Lett..

[151] Yan J., Wan Y., Ji Z., Li C., Tao C., Tang Y., Zhang Y., Liu Y., Liu J. (2023). Motor neuron‐specific membrane depolarization of transected peripheral nerves by upconversion nanoparticle‐Mediated optogenetics. Adv. Funct. Mater..

[152] Sun Y., Feng W., Yang P., Huang C., Li F. (2015). The biosafety of lanthanide upconversion nanomaterials. Chem. Soc. Rev..

[153] Liu Z., Ju E., Liu J., Du Y., Li Z., Yuan Q., Ren J., Qu X. (2013). Direct visualization of gastrointestinal tract with lanthanide-doped BaYbF5 upconversion nanoprobes. Biomaterials.

[154] Hlaváček A., Farka Z., Mickert M.J., Kostiv U., Brandmeier J.C., Horák D., Skládal P., Foret F., Gorris H.H. (2022). Bioconjugates of photon-upconversion nanoparticles for cancer biomarker detection and imaging. Nat. Protoc..

[155] Xu Z., Wang C., Ma R., Sha Z., Liang F., Sun S. (2022). Aptamer-based biosensing through the mapping of encoding upconversion nanoparticles for sensitive CEA detection. Analyst.

[156] Zhou L., Wang R., Yao C., Li X., Wang C., Zhang X., Xu C., Zeng A., Zhao D., Zhang F. (2015). Single-band upconversion nanoprobes for multiplexed simultaneous in situ molecular mapping of cancer biomarkers. Nat. Commun..

[157] Jayakumar M.K., Idris N.M., Zhang Y. (2012). Remote activation of biomolecules in deep tissues using near-infrared-to-UV upconversion nanotransducers. Proc. Natl. Acad. Sci. U. S. A..

[158] Tian Y., Zhang Y., Zhang X., Pan H., Zhang L., Liu S., Chen Y., Su L., Zhao P., Chang J., Wang H. (2022). "Magnetism-Optogenetic" system for wireless and highly sensitive neuromodulation. Adv. Healthcare Mater..

[159] Zhang Y., Zhang X., Wang H., Tian Y., Pan H., Zhang L., Wang F., Chang J. (2020). Remote regulation of optogenetic proteins by a magneto‐luminescence microdevice. Adv. Funct. Mater..

[160] Wu X., Zhu X., Chong P., Liu J., Andre L.N., Ong K.S., Brinson K., Mahdi A.I., Li J., Fenno L.E., Wang H., Hong G. (2019). Sono-optogenetics facilitated by a circulation-delivered rechargeable light source for minimally invasive optogenetics. Proc. Natl. Acad. Sci. U. S. A..

[161] Wang W., Wu X., Kevin Tang K.W., Pyatnitskiy I., Taniguchi R., Lin P., Zhou R., Capocyan S.L.C., Hong G., Wang H. (2023). Ultrasound-triggered in situ photon emission for noninvasive optogenetics. J. Am. Chem. Soc..

[162] Su Y., Walker J.R., Park Y., Smith T.P., Liu L.X., Hall M.P., Labanieh L., Hurst R., Wang D.C., Encell L.P., Kim N., Zhang F., Kay M.A., Casey K.M., Majzner R.G., Cochran J.R., Mackall C.L., Kirkland T.A., Lin M.Z. (2020). Novel NanoLuc substrates enable bright two-population bioluminescence imaging in animals. Nat. Methods.

[163] Dou Y., Chen R., Liu S., Lee Y.T., Jing J., Liu X., Ke Y., Wang R., Zhou Y., Huang Y. (2023). Optogenetic engineering of STING signaling allows remote immunomodulation to enhance cancer immunotherapy. Nat. Commun..

[164] Zheng B., Wang H., Pan H., Liang C., Ji W., Zhao L., Chen H., Gong X., Wu X., Chang J. (2017). Near-infrared light triggered upconversion optogenetic nanosystem for cancer therapy. ACS Nano.

[165] Zhu X., Chen S., Hu X., Zhao L., Wang Y., Huang J., Chen J., Qiu Y., Zhang X., Wang M., Yang X., Zhang Y., Zhu Y. (2023). Near-infrared nano-optogenetic activation of cancer immunotherapy via engineered bacteria. Adv. Mater..

[166] Yu Y., Wu X., Wang M., Liu W., Zhang L., Zhang Y., Hu Z., Zhou X., Jiang W., Zou Q., Cai F., Ye H. (2022). Optogenetic-controlled immunotherapeutic designer cells for post-surgical cancer immunotherapy. Nat. Commun..

[167] Ding J., Lu J., Zhang Q., Xu Y., Song B., Wu Y., Shi H., Chu B., Wang H., He Y. (2023). Camouflage nanoparticles enable in situ bioluminescence-driven optogenetic therapy of retinoblastoma. ACS Nano.

[168] Zhang Y., Xue X., Fang M., Pang G., Xing Y., Zhang X., Li L., Chen Q., Wang Y., Chang J., Zhao P., Wang H. (2022). Upconversion optogenetic engineered bacteria system for time-resolved imaging diagnosis and light-controlled cancer therapy. ACS Appl. Mater. Interfaces.

[169] Wu C., Cui M., Cai L., Chen C., Zhu X., Wu Y., Liu J., Wang H., Zhang Y. (2022). NIR-responsive photodynamic nanosystem combined with antitumor Immune optogenetics bacteria for precise synergetic therapy. ACS Appl. Mater. Interfaces.

[170] Qiao L., Niu L., Wang Z., Deng Z., Di D., Ma X., Zhou Y., Kong D., Wang Q., Yin J., Jin L., Sun J., Feng B., Lu W., Cai F., Guan N., Ye H. (2025). Engineered bacteria for near-infrared light-inducible expression of cancer therapeutics. Nat. Cancer.

[171] Chen X., Zhang X., Liu Y., Chen Y., Zhao Y. (2024). Upconversion nanoparticle-anchored metal-organic framework nanostructures for remote-controlled cancer optogenetic therapy. J. Am. Chem. Soc..

[172] Ren X., Lin J., Wang X., Liu X., Meng E., Zhang R., Sang Y., Zhang Z. (2017). Photoactivatable RNAi for cancer gene therapy triggered by near-infrared-irradiated single-walled carbon nanotubes. Int. J. Nanomed..

[173] Jaeger M., Anastasio A., Chamy L., Brustlein S., Vincentelli R., Durbesson F., Gigan J., Thépaut M., Char R., Boussand M., Lechelon M., Argüello R.J., Marguet D., He H.T., Lasserre R. (2023). Light-inducible T cell engagers trigger, tune, and shape the activation of primary T cells. P Natl. Acad. Sci. USA.

[174] Kuwasaki Y., Suzuki K., Yu G.G., Yamamoto S., Otabe T., Kakihara Y., Nishiwaki M., Miyake K., Fushimi K., Bekdash R., Shimizu Y., Narikawa R., Nakajima T., Yazawa M., Sato M. (2022). A red light-responsive photoswitch for deep tissue optogenetics. Nat. Biotechnol..

[175] Redchuk T.A., Omelina E.S., Chernov K.G., Verkhusha V.V. (2017). Near-infrared optogenetic pair for protein regulation and spectral multiplexing. Nat. Chem. Biol..

[176] Schmidl S.R., Ekness F., Sofjan K., Daeffler K.N., Brink K.R., Landry B.P., Gerhardt K.P., Dyulgyarov N., Sheth R.U., Tabor J.J. (2019). Rewiring bacterial two-component systems by modular DNA-binding domain swapping. Nat. Chem. Biol..

[177] Kasatkina L.A., Ma C., Sheng H., Lowerison M., Menozzi L., Baloban M., Tang Y., Xu Y., Humayun L., Vu T., Song P., Yao J., Verkhusha V.V. (2025). Deep-tissue high-sensitivity multimodal imaging and optogenetic manipulation enabled by biliverdin reductase knockout. Nat. Commun..

[178] Wang X., Kang L., Kong D., Wu X., Zhou Y., Yu G., Dai D., Ye H. (2024). A programmable protease-based protein secretion platform for therapeutic applications. Nat. Chem. Biol..

[179] Dou Y.L., Chen R., Liu S.Y., Lee Y.T., Jing J., Liu X.X., Ke Y.P., Wang R., Zhou Y.B., Huang Y. (2023). Optogenetic engineering of STING signaling allows remote immunomodulation to enhance cancer immunotherapy. Nat. Commun..

[180] Cheng Y., Sun R., He M., Zhang M., Hou X., Sun Y., Wang J., Xu J., He H., Wang H., Lan M., Zhao Y., Yang Y., Chen X., Gao F. (2022). Light-switchable diphtherin transgene system combined with losartan for triple negtative breast cancer therapy based on nano drug delivery system. Int. J. Pharm..

[181] He K., Jiang H., Zhang W., Yang N., Li S., Wang Y., Zhang J., Li X., Tan L., Yang G., Li H., Lu Y. (2025). Optogenetic engineered macrophages for light-induced M1 polarization and enhanced chemo-immunotherapy in melanoma models. Exp. Cell Res..

[182] Qiao S., Xin F., Wu M., Zheng Y., Zhao B., Zhang C., Liu X., Wei Z., Liu J. (2021). A remotely controlled NIR-II photothermal-sensitive transgene system for hepatocellular carcinoma synergistic therapy. J. Mater. Chem. B.

[183] Cheng Y., Zou J., He M., Hou X., Wang H., Xu J., Yuan Z., Lan M., Yang Y., Chen X., Gao F. (2024). Spatiotemporally controlled pseudomonas exotoxin transgene system combined with multifunctional nanoparticles for breast cancer antimetastatic therapy. J. Contr. Release.

[184] Kasatkina L.A., Ma C., Matlashov M.E., Vu T., Li M., Kaberniuk A.A., Yao J., Verkhusha V.V. (2022). Optogenetic manipulation and photoacoustic imaging using a near-infrared transgenic mouse model. Nat. Commun..

[185] Chow B.Y., Han X., Dobry A.S., Qian X., Chuong A.S., Li M., Henninger M.A., Belfort G.M., Lin Y., Monahan P.E., Boyden E.S. (2010). High-performance genetically targetable optical neural silencing by light-driven proton pumps. Nature.

